# Structure and functional analysis of the *Legionella pneumophila* chitinase ChiA reveals a novel mechanism of metal-dependent mucin degradation

**DOI:** 10.1371/journal.ppat.1008342

**Published:** 2020-05-04

**Authors:** Saima Rehman, Lubov S. Grigoryeva, Katherine H. Richardson, Paula Corsini, Richard C. White, Rosie Shaw, Theo J. Portlock, Benjamin Dorgan, Zeinab S. Zanjani, Arianna Fornili, Nicholas P. Cianciotto, James A. Garnett

**Affiliations:** 1 Centre for Host-Microbiome Interactions, Dental Institute, King’s College London, London, United Kingdom; 2 Department of Microbiology and Immunology, Feinberg School of Medicine, Northwestern University, Chicago, Illinois, United States of America; 3 Chemistry and Biochemistry Department, School of Biological and Chemical Sciences, Queen Mary University of London, London, United Kingdom; Purdue University, UNITED STATES

## Abstract

Chitinases are important enzymes that contribute to the generation of carbon and nitrogen from chitin, a long chain polymer of N-acetylglucosamine that is abundant in insects, fungi, invertebrates and fish. Although mammals do not produce chitin, chitinases have been identified in bacteria that are key virulence factors in severe respiratory, gastrointestinal and urinary diseases. However, it is unclear how these enzymes are able to carry out this dual function. *Legionella pneumophila* is the causative agent of Legionnaires' disease, an often-fatal pneumonia and its chitinase ChiA is essential for the survival of *L*. *pneumophila* in the lung. Here we report the first atomic resolution insight into the pathogenic mechanism of a bacterial chitinase. We derive an experimental model of intact ChiA and show how its N-terminal region targets ChiA to the bacterial surface after its secretion. We provide the first evidence that *L*. *pneumophila* can bind mucins on its surface, but this is not dependent on ChiA. This demonstrates that additional peripheral mucin binding proteins are also expressed in *L*. *pneumophila*. We also show that the ChiA C-terminal chitinase domain has novel Zn^2+^-dependent peptidase activity against mammalian mucin-like proteins, namely MUC5AC and the C1-esterase inhibitor, and that ChiA promotes bacterial penetration of mucin gels. Our findings suggest that ChiA can facilitate passage of *L*. *pneumophila* through the alveolar mucosa, can modulate the host complement system and that ChiA may be a promising target for vaccine development.

## Author summary

A broad range of organisms produce chitinase enzymes that digest chitin, the second most abundant carbohydrate on earth. Chitinases have also been identified that are important factors in major bacterial diseases but it is unclear how. *Legionella pneumophila* causes Legionnaires’ disease, a severe form of pneumonia, and its chitinase ChiA is essential for the survival of *L*. *pneumophila* during infection of the lung. Using structural biology and microbiology methods we have determined that ChiA can associate with the *L*. *pneumophila* surface and along with other outer membrane proteins can also bind mammalian mucins. We also identified a new and unique enzyme mechanism where *L*. *pneumophila* ChiA can hydrolyse the peptide bonds of mucin-like proteins. Mucins are major components of the mucous that lines the surface of the respiratory, digestive, and urogenital tracts and acts as a first line of defence during infection. This is the first mechanistic understanding of how a chitinase can promote disease through additional peptidase activity and suggests that *L*. *pneumophila* ChiA can modulate host immune responses and disperse host mucosa during infection.

## Introduction

*Legionella pneumophila* is a Gram-negative bacterium that can withstand large variation in pH and temperature. When humans are exposed to *L*. *pneumophila*, it can infect macrophages and epithelia in the lungs and trigger chronic inflammation and tissue damage [1]. *L*. *pneumophila* is the causative agent of Legionnaires’ disease, an often-fatal pneumonia, and Pontiac fever, a milder flu-like disease, and rates of infection are increasing each year [1–4]. Although infection is primarily via inhalation of contaminated water droplets from aerosolizing devices [5], there is also now evidence for person-to-person transmission [6, 7].

Upon invasion of eukaryotic hosts, *L*. *pneumophila* avoids fusion with canonical endosomal/lysosomal pathways by forming a membrane bound compartment, the *Legionella* containing vacuole (LCV) [1]. *L*. *pneumophila* exports over 300 proteins from this modified phagosome into the host cytoplasm by the Icm/Dot type IVb secretion system [1]. These effectors manipulate host signalling pathways and mediate evasion of the host’s degradative lysosomal pathway, enabling *L*. *pneumophila* to replicate to large numbers [8]. *L*. *pneumophila* also expresses a type II secretion system (T2SS) [9], which secretes at least 25 proteins, including almost 20 enzymes and substrates that contain a high proportion of unique amino acid sequence with no homology outside of the *Legionella* genus [10, 11]. The T2SS is important for both intracellular and extracellular lifestyles [10]. These processes include extracellular growth at low temperatures, biofilm formation, intracellular replication in amoebae and macrophages, dampening of cytokine output from infected cells and persistence in lungs [10, 12–18].

Among the *L*. *pneumophila* type II substrates, ChiA is an 81 kDa endochitinase with a novel amino acid sequence at its N-terminus and a putative glycosyl hydrolase 18 (GH18) domain at its C-terminus [17]. Chitin is an insoluble carbohydrate composed of linear β-1,4-linked N-acetylglucosamine (GlcNAc) residues and its degradation by chitinase enzymes serves as an important source of nutrients for many bacteria [19]. Chitin is not synthesized by mammals and *L*. *pneumophila chiA* mutants are not impaired for growth in *Acanthamoeba castellanii*, *Vermamoeba vermiformis*, and *Willaertia magna* amoebae, macrophage-like U937 cells, and lung epithelial cell-like A549 cells [14, 17, 20, 21]. However, they are less able to survive in the lungs of A/J mice, suggesting that ChiA is required for optimal survival of *L*. *pneumophila* in the lungs [17], although how it is able to promote infection is unknown. ChiA is present within 53% of *Legionella* species, and its closest homologs outside of the *Legionella* genus are within other γ-Proteobacteria, especially species of *Aquicella* that, like *Legionella*, infect amoebae [10]. Interestingly, *L*. *pneumophila* ChiA also has high relatedness to proteins encoded by both mimiviruses that infect amoebae and water moulds, raising the possibility that ChiA was acquired by inter-kingdom horizontal gene transfer [10].

In this study, we report a structural model for full-length ChiA based on X-ray crystallographic, template based modelling and small angle X-ray scattering (SAXS) data. ChiA is composed of four domains (N1, N2, N3 and CTD, from N- to C-terminus) which have structural homology to those associated with other chitinase enzymes. Using chitin binding and chitinase activity assays, we show that ChiA-N1 is a chitin binding module and we confirm that ChiA-CTD is a glycosyl hydrolase domain. Using binding assays, we show that both the ChiA-N3 domain and eukaryotic mucins can associate with the *L*. *pneumophila* surface. Lastly, our structural and biochemical studies demonstrate that ChiA-CTD has novel peptidase activity against mucin-like glycoproteins, which is independent of its chitinase active site. Our work provides novel molecular insight into the virulence mechanism of a bacterial chitinase and suggests that ChiA has a role in modulating host immune responses and facilitates *L*. *pneumophila* penetration of the alveolar mucosa during infection.

## Results

### ChiA is a multi-domain protein

**Fig 1:**
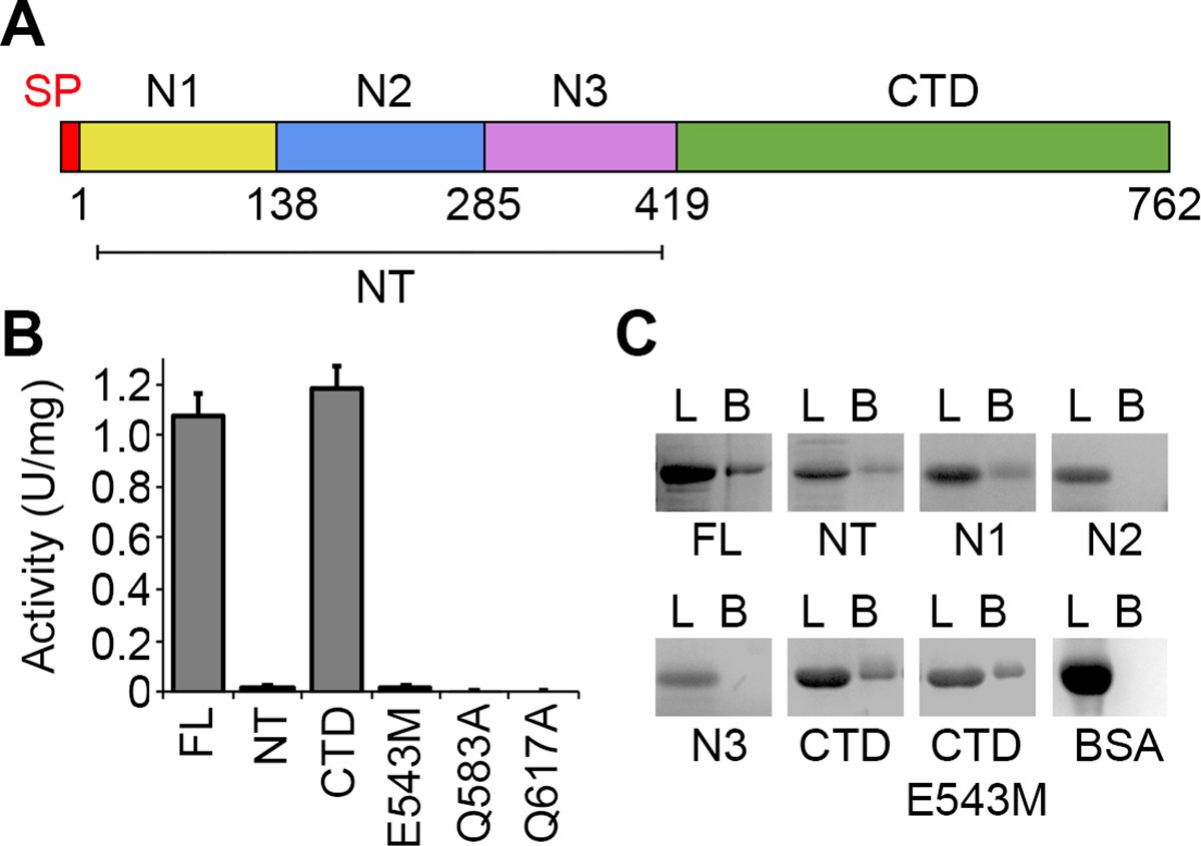
Chitin binding and endochitinase functions of ChiA. (A) Schematic representation of ChiA with domain boundaries annotated. (B) ChiA-FL, subdomains (NT, N1, N2, N3, CTD) and ChiA-CTD mutants (E543M, Q583A, Q617A) were assayed for chitinase activity against *p*-NP-[GlcNAc]_3_. Data represent the mean and standard deviation for triplicate experiments. (C) Chitin pull-down experiment to assess direct interactions between immobilized chitin and ChiA. ChiA-FL and subdomains (NT, N1, N2, N3, CTD, CTD^E543M^) were incubated with chitin beads and analysed by SDS-PAGE. BSA was used as a control. L: loaded sample; B: eluted beads. Eluted samples undergo an upward shift compared to the input sample due to differences in buffer conditions. Data is representative of three independent repeat experiments.

Full-length ChiA from *L*. *pneumophila* 130b (ChiA-FL; numbered 1–762 for the mature protein; NCBI accession WP_072401826.1) with an N-terminal His_6_-tag was produced in *Escherichia coli* K12 and purified by nickel-affinity and size exclusion chromatography. Despite extensive screening ChiA-FL resisted crystallization and we therefore used bioinformatics analysis to produce a series of subdomain constructs for further characterization (Fig 1A). While previous examination of the C-terminal domain of ChiA (ChiA-CTD: residues 419–762) had revealed high primary sequence homology to other GH18 chitinase domains [17], the N-terminal region (ChiA-NT; residues 1–417) contains unique primary sequence with no significant homology to any other known protein. Nonetheless, through secondary structure prediction [22] and template based modelling using the Phyre2 [23] and Robetta [24] servers, we identified three putative N-terminal subdomains based on predicted structural similarity with carbohydrate-binding modules (CBMs; ChiA-N1: residues 1–140), fibronectin type-III-like domains (Fn3; ChiA-N2: residues 138–299) and a chitinase A N-terminal domain (ChiN; ChiA-N3: residues 285–417) (Fig 1A and S1 Table).

To examine the function of the ChiA N-terminal domains we began by examining their endochitinase activity [17]. Each construct was expressed with an N-terminal His_6_-tag in *E*. *coli* K12 and purified by nickel-affinity and size exclusion chromatography. All reagents were well folded as determined by 1D ^1^H nuclear magnetic resonance (NMR) spectroscopy (S1 Fig). As expected, ChiA-FL and ChiA-CTD were both active against *p*-nitrophenyl β-D-*N*,*N′*,*N″* triacetylchitotriose (*p*NP-[GlcNAc]_3_) but no activity was detected for ChiA-NT or an E543M ChiA-CTD active site mutant (ChiA-CTD^E543M^) (Fig 1B and S2 Fig). We then assayed binding of ChiA sub-domains to immobilized chitin and observed that in addition to ChiA-CTD, the N1-domain also recognizes chitin polymers, which supports its role as a carbohydrate binding module (Fig 1C).

### Atomic structure of ChiA-CTD

**Fig 2:**
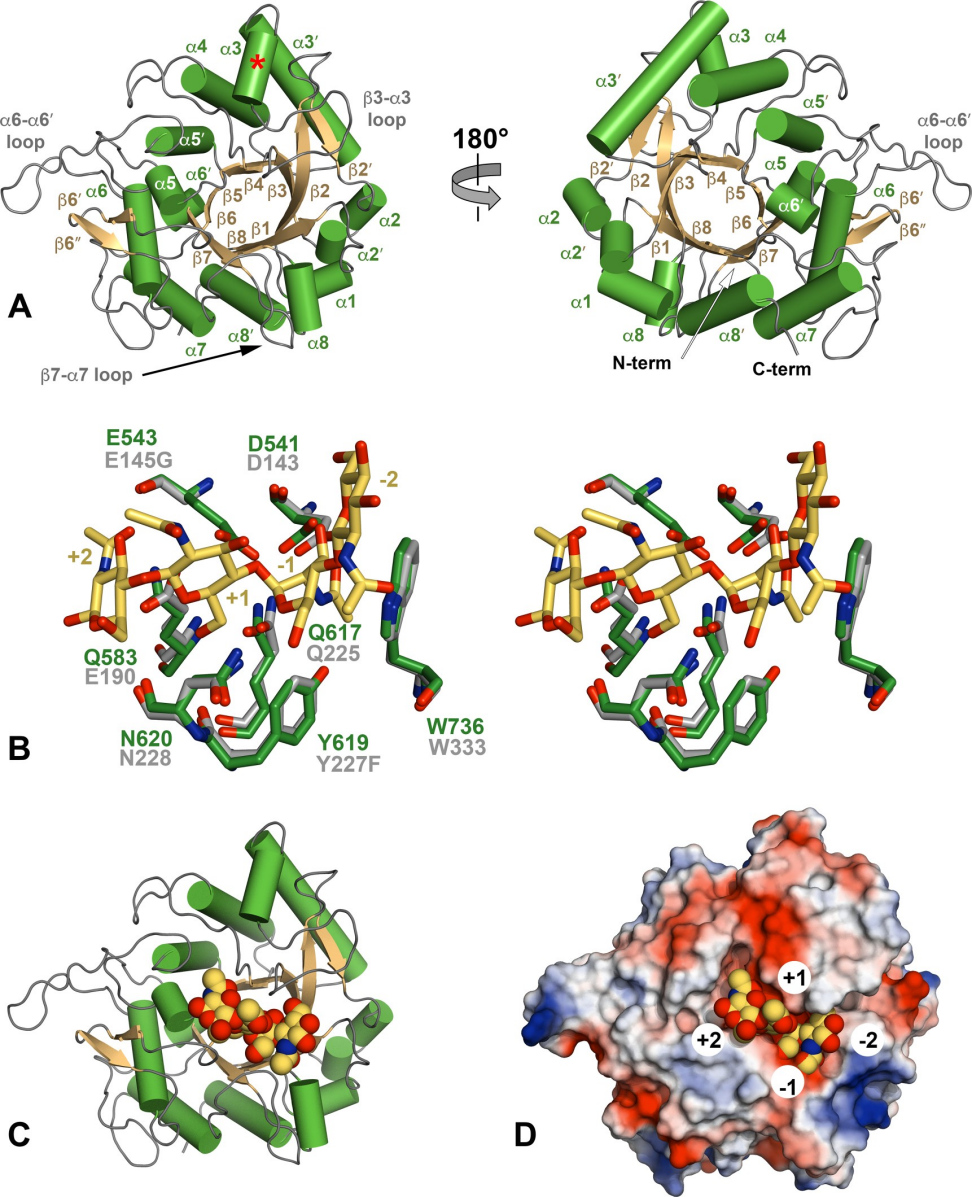
Crystal structure of ChiA-CTD. (A) Cartoon representation of ChiA-CTD with secondary structure and extended loops annotated. Additional ChiA-CTD α3-helix is highlighted with a red asterisk. (B) Stick representation of the ChiA-CTD active site (green) superimposed with ChiNCTU2 E145G/Y227F mutant (grey) in complex with chitotetraose (yellow) (PDB ID code 3n18) [27]. Mutated residues in ChiNCTU2 are indicated and carbohydrate positions relative to the hydrolysed glycosidic bond are numbered. (C) Model of ChiA-CTD shown as cartoon and (D) electrostatic surface potential, bound to chitotetraose drawn as spheres.

We next initiated crystallographic studies of the ChiA subdomains. We readily obtained crystals for ChiA-CTD and its structure was determined using iodide single isomorphous replacement with anomalous scattering (I-SIRAS) phasing. Electron-density maps were refined to 1.7 Å (Table 1) and the final model contains two identical chains, with all molecules built except for the N-terminal His_6_-tags and adjacent ChiA-CTD residues Val419 to Gly424. Each chain forms an anticipated GH18 α/β-fold and is composed of 11 β-strands and 13 α-helices (Fig 2A). High concentrations of 2-methyl-2,4-pentanediol (MPD) were used as a precipitant during crystallization and we observed four MPD molecules in the final model; one bound to the catalytic Asp541 and Glu543 residues (MPD-1) and one within a hydrophobic pocket formed by the α5 helix and α5-α5′/β5-β6′ loops (MPD-2) (S3 Fig).

**Table 1:** Diffraction data and refinement statistics.

	Native	Iodide
Crystal Parameters		
Space group	*C*2	*C*2
Cell dimensions (Å; º)	*a* = 97.37, *b* = 56.64, *c* = 128.96; β = 93.79	*a* = 97.25, *b* = 57.35, *c* = 129.42; β = 93.43
Data collection		
Beamline	DLS I04	DLS I02
Wavelength (Å)	0.97949	1.70000
Resolution (Å)	64.34–1.71 (1.75–1.71)	64.59–1.89 (1.94–1.89)
Unique observations	75905 (5555)	52043 (2309)
R_sym_	0.061 (0.658)	0.189 (3.697)
<*I*>/*σI*	19.2 (2.8)	9.7 (1.4)
Completeness (%)	99.9 (99.9)	91.2 (55.0)
Redundancy	6.8 (6.9)	13.1 (11.4)
Wilson B (Å^2^)	19.3	20.9
Phasing		
Figure of merit (acentric/centric)	-	0.159/0.139
Phasing power isotropic (acentric/centric)	-	1.059/0.919
Phasing power anomalous	-	0.887
Refinement		
R_work_/R_free_ (%)	14.6/16.8	-
Number of protein residues	678	-
Number of water molecules	667	-
Number of ligands	2 MPD, 2 MRD	-
<*B*> protein	22.2	-
<*B*> waters	35.8	-
<*B*> ligands	21.6	-
rmsd stereochemistry		
Bond lengths (Å)	0.008	-
Bond angles (°)	1.472	-
Ramachandran analysis		
Residues in outlier regions (%)	0.0	-
Residues in favoured regions (%)	95.9	-
Residues in allowed regions (%)	100	-

Numbers in parentheses refer to the outermost resolution shell.

*R*_sym_ = Σ|*I*–<*I*>|/Σ*I* where *I* is the integrated intensity of a given reflection and <*I*> is the mean intensity of multiple corresponding symmetry-related reflections.

*R*_work_ = Σ||F_o_|–|F_c_||/ΣF_o_ where F_o_ and F_c_ are the observed and calculated structure factors, respectively.

*R*_free_ = *R*_work_ calculated using 10% random data excluded from the refinement.

rmsd stereochemistry is the deviation from ideal values.

Ramachandran analysis was carried out using Molprobity [25].

The overall structure of ChiA-CTD is highly similar to other GH18 chitinase domains, and the Dali server [26] identified *Bacillus cereus* ChiNCTU2 enzyme inactive E145G/Y227F mutant in complex with chitotetraose (Protein Data Bank (PDB) ID code 3n18) [27]; *Bacillus anthracis* Chi36 (PDB ID code 5kz6); *Chromobacterium violaceum* ChiA (PDB ID code 4tx8); and *Streptomyces coelicolor* ChiA (PDB ID code 3ebv) as having the highest homologies (Z score: 36.3, 35.9, 34.4, 34.1 respectively; rmsd: 2.2 Å, 2.3 Å, 2.2 Å, 1.8 Å, respectively). The chitinase active sites of ChiNCTU2 and ChiA-CTD have high primary sequence identity and tertiary structure homology (Fig 2B and S4 Fig) and modelling of chitotetraose binding indicates that chitin lines a negatively charged valley on the surface of ChiA-CTD (Fig 2C and 2D). Chitotetraose overlays with MPD-1 in the active site (S3 Fig) and the MPD-2 site is positioned adjacent to the reducing end of the modelled chitotetraose. In this binding model, Gln583 and Gln617 have a central role in the correct positioning of chitotetraose in the ChiA-CTD active site. We therefore created Q583A and Q617A mutants in recombinant ChiA-CTD and as anticipated, these mutants showed no activity against *p*NP-[GlcNAc]_3_ (Fig 1B and S2 Fig). However, *L*. *pneumophila* ChiA-CTD also possesses unique features that are not observed in homologous structures. These include an additional α-helix (α3), an extended β3-α3 loop, an extended α6-α6′ loop and an extended β7-α7 loop (Fig 2A and S4 and S5 Figs).

### ChiA is an elongated and dynamic structure in solution

We used small angle X-ray scattering (SAXS) to model the global structure of full-length ChiA in solution. Four different concentrations at 4, 2, 1, and 0.5 mg/ml were measured but signs of aggregation were apparent at concentrations above 1 mg/ml (S6 Fig). To achieve the highest signal/noise ratio, all further analysis was carried out with the data from the 1 mg/ml sample (S6 Fig and S2 Table). Guinier analysis suggested a radius of gyration (*R*_g_), the root mean square distance to the particles centre of mass, of 5.43 nm and analysis of the distance distribution function (P(r)) suggested a maximum particle dimension (*D*_max_) of 17.77 nm and *R*_g_ of 5.45 nm (S6 Fig). Using BSA as a standard, we calculated a particle molecular mass of 89.2 kDa, which is within the method error range for a monomeric 82.6 kDa ChiA.

**Fig 3:**
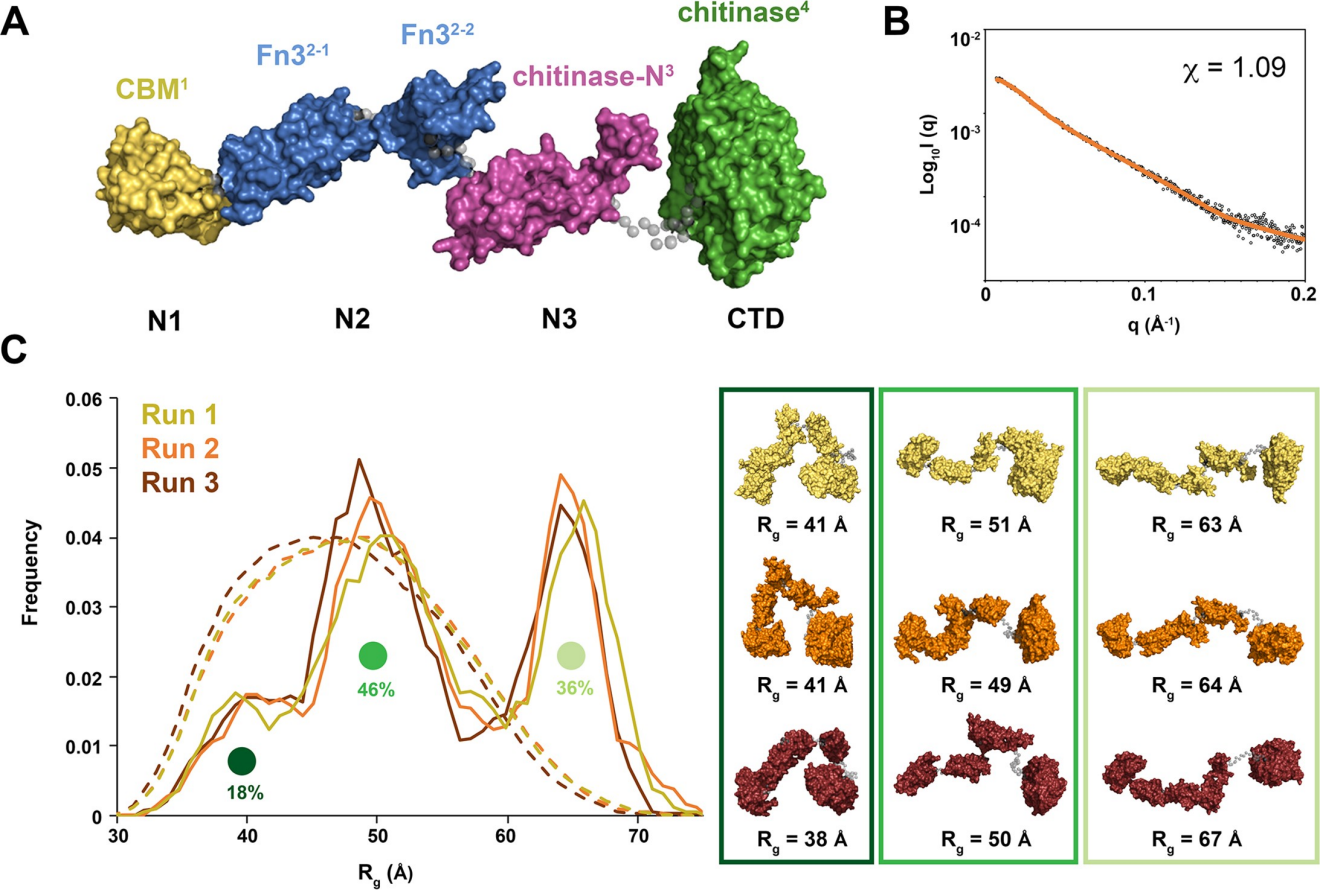
Model of ChiA-FL in solution. (A) Initial model of ChiA generated by EOM 2.0 [30]. Linkers are shown as grey spheres. (B) EOM fit (orange line) to the ChiA SAXS data (black open circles) with χ^2^ of 1.09. (C) Three independent ensemble optimization method runs (yellow, orange and burgundy) yielded similar distributions of three populations. Sample ChiA models corresponding to the centre of each population for all three runs are shown.

Kratky, Kratky-Debye and Porod-Debye plot analyses of the SAXS data indicated that ChiA is a highly dynamic particle in solution (S6 Fig) [28]. This is likely due to flexibility within the ChiA inter-domain linkers and we therefore used the ensemble optimization method (EOM) to determine molecular model ensembles of ChiA that best fit the SAXS data [29]. As we were not able to obtain crystals for ChiA N-domains, an initial model of ChiA was created using a Phyre2 derived N1-domain (residues 22–147), a Robetta derived N2-domain (encompassing two further subdomains: residues 152–245 and 248–305), a Phyre2 derived ChiA N3-domain (residues 315–414) and the crystal structure of ChiA-CTD (residues 439–777), separated by flexible linkers (Fig 3A and S1 Table) [23, 24, 29]. Ensemble optimization analysis of the scattering data yielded an excellent fit between experimental and calculated SAXS profiles (χ^2^: 1.1), which again indicates that ChiA is highly flexible in solution (R_flex_ 91.4) [30] with conformation ensembles clustered within three populations (Fig 3B and 3C and S3 Table). The majority of the simulated conformations exhibited partially extended or fully extended structures at *Rg* 44–56 Å or *Rg* 60–71 Å, respectively, whereas minor conformations of closed structures were also populated at *Rg* 33–43 Å. This analysis provides experiential support for the global features of our ChiA N-domain models but also suggests that there is cooperation between ChiA domains. As inter-domain flexibility is a key feature of processive enzymes [31], these data suggest that processivity may also be important for the function of ChiA.

### ChiA is targeted to the *L*. *pneumophila* surface

**Fig 4:**
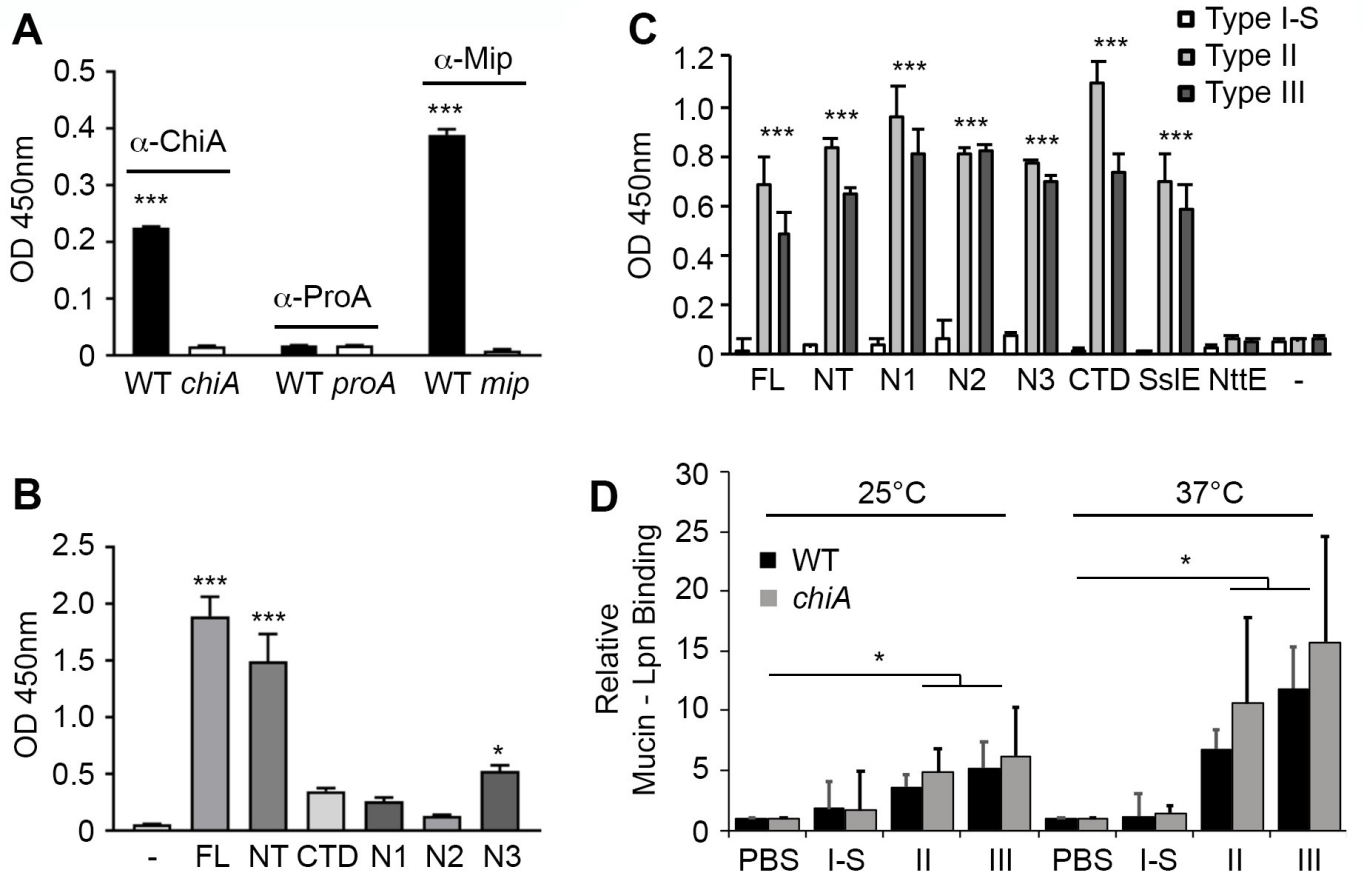
*L*. *pneumophila* surface association of ChiA and mucin binding. (A) Whole cell ELISA of *L*. *pneumophila* wild-type 130b (WT), *chiA* mutant NU318 (*chiA*), *proA* mutant AA200 (*proA*) and *mip* mutant NU203 (*mip*) detected with antiserum specific for either ChiA, ProA, or Mip. (B) Whole cell ELISA of *chiA* mutant incubated with either recombinant ChiA-FL or subdomains (NT, N1, N2, N3, CTD) and detected with antiserum specific for ChiA. PBS buffer alone was used as a control (-). Multiple comparisons against the control were made using a one-way ANOVA, *Holm*-*Šídák* multiple comparisons test; * *P* < 0.05, *** *P* < 0.001. (C) ELISA analysis of binding between immobilised type I-S, II or III porcine stomach mucins and His-tagged ChiA-FL, subdomains (NT, N1, N2, N3, CTD) and controls (SslE, NttE) detected with anti-His-tag antibody. BSA-coated wells were used as controls. *** *P* < 0.001; verses control empty well by two-tailed Student’s test. (D) Mucin binding to GFP-expressing *L*. *pneumophila* WT or *chiA* mutant strains were incubated at 25°C or 37°C with PBS, type I-S, II or III mucins followed by Texas Red-tagged wheat germ agglutinin (WGA). Mucin binding to bacteria was quantified by flow cytometry. * *P* < 0.05; verses PBS control by two-tailed Student’s test. All data represent the mean and standard deviation for triplicate experiments.

Although we have repeatedly detected secreted ChiA in bacterial culture supernatants [17, 32], it has been well documented in other bacteria that some type II substrates associate with the bacterial surface upon their secretion [11, 33]. To determine whether ChiA is also targeted to the bacterial surface, *L*. *pneumophila* 130b was examined by whole-cell ELISA using anti-ChiA, anti-Mip and anti-ProA antibodies [32, 34] (Fig 4A). Both ChiA and Mip, a known surface exposed protein [35], were positive by ELISA, whilst the metalloprotease ProA, another T2SS substrate [10, 36], was negative. As the translocation signal for ChiA (and T2SS substrates in general) has yet to be determined [10, 11], *in vivo* truncations or site directed mutagenesis of *chiA* could inadvertently impede the secretion of ChiA. Therefore, to examine which region of ChiA is responsible for this localization, a *L*. *pneumophila* NU318 (*chiA*) mutant [17] was incubated with recombinant ChiA fragments and the whole-cell ELISA was repeated using ChiA antisera (Fig 4B). ChiA-NT and the N3-domain exhibited significant binding to the bacterial surface while no binding was observed for ChiA-CTD, ChiA-N1 or ChiA-N2. A direct comparison between anti-ChiA antibodies binding to ChiA-FL, ChiA-NT and ChiA-CTD indicated that the antiserum binds to the N- and C-terminal fragments at a similar or higher level than to full length ChiA (S7 Fig). Likewise, direct comparison between the three N-terminal subdomains revealed that anti-ChiA antibodies bind to the N1 and N2 subdomains with comparable or higher affinity as they do to ChiA-N3 (S7 Fig). Thus, the greater association of ChiA-FL, ChiA-NT and ChiA-N3 with the *L*. *pneumophila* surface, as detected by whole-cell ELISA (Fig 4B), is not simply the result of a reduced ability of the antiserum to recognize ChiA-CTD, ChiA-N1, and ChiA-N2.

### ChiA is a mucin binding protein

Some bacterial chitinases and chitin binding proteins are able to promote infection through adhesion to and/or degradation of host glycoconjugates [37] and we hypothesized that ChiA may interact with exogenous mucins in the lungs and elsewhere. We therefore examined the binding capacity of recombinant ChiA-FL, ChiA domains, *L*. *pneumophila* NttE, another T2SS substrate [10, 17], and *E*. *coli* SslE, a known mucin binding protein and mucinase [38], to immobilized commercially available mucin extracts by ELISA using anti-His antibodies (Fig 4C). All ChiA samples and SslE displayed significant adhesion to mucins isolated from porcine stomachs (type II and III), but this was not observed with a mucin extract from bovine submaxillary glands (type I-S). Conversely, NttE showed no binding to any of the mucin samples. This confirmed that ChiA has additional specificity for non-chitinous ligands and implied that ChiA could bind host glycoproteins on the *Legionella* surface. To assess this, we incubated *L*. *pneumophila* 130b wild-type and NU318 (*chiA*) mutant strains with type I-S, II and III mucin extracts followed by wheat germ agglutinin and measured their binding to the bacterial surface by flow cytometry. Whether examined at 25°C or 37°C, type II and III extracts showed strong association to both *L*. *pneumophila* strains, but submaxillary gland mucins did not (Fig 4D). However, there was no significant difference in binding of the type II and III mucins between wild-type and *chiA* mutant strains, which indicates that other factors apart from ChiA are present on the *Legionella* surface that can also recognize mucins and in the case of the mutant, compensate for the loss of ChiA. Interestingly, the mean binding of *L*. *pneumophila* to both type II and III mucins trends slightly higher in the *chiA* mutant strain and we speculated that this may be due to ChiA degrading mucins on the bacterial surface on the wild-type strain.

### ChiA increases penetration of *L*. *pneumophila* through the mucin layer

**Fig 5:**
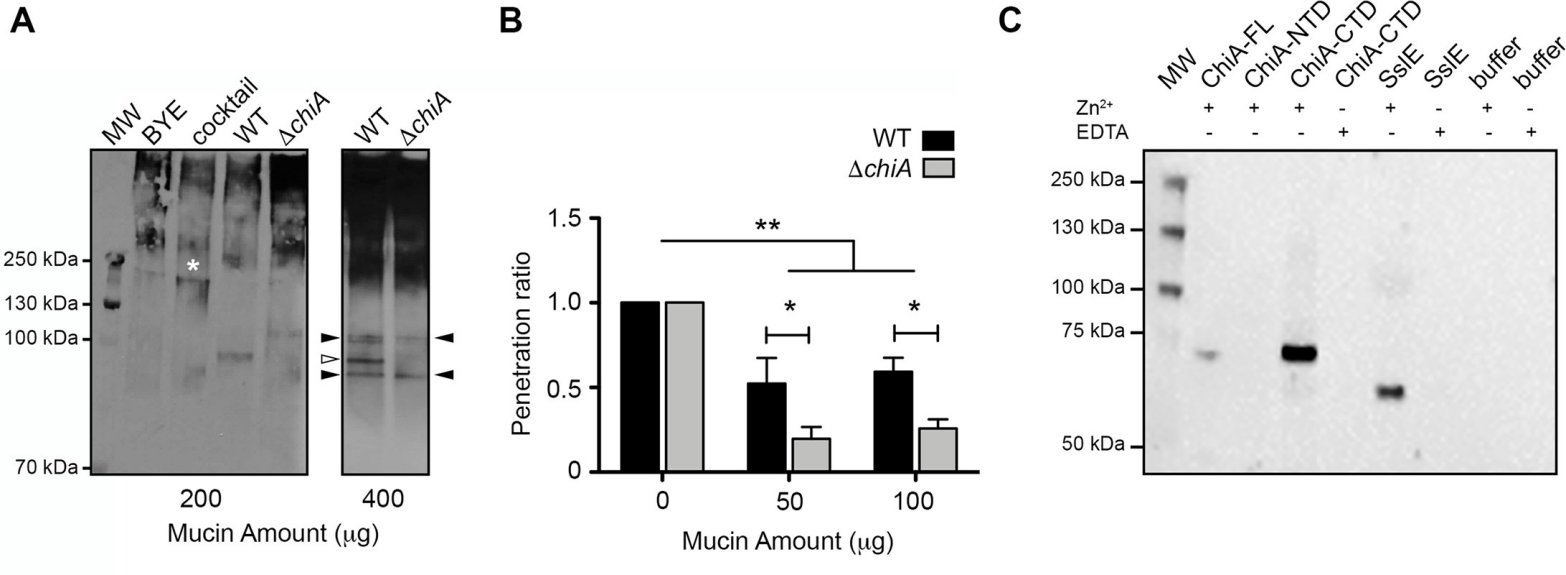
Mucinase activity of ChiA. (A) Secreted mucinase activity of *L*. *pneumophila* wild-type and *chiA* mutant strains. Left panel: immunoblot of type II porcine stomach mucins (200 μg) incubated with either BYE medium alone (BYE), a cocktail of known mucinase enzymes added to BYE medium (cocktail), or supernatants from BYE cultures of wild-type 130b (WT) or *chiA* mutant NU318 (Δ*chiA*). Asterisk highlights a lower-MW (~200 kDa) mucin species generated by the cocktail that is not present in the supernatant samples. Right panel: immunoblot of type II porcine stomach mucins (400 μg) incubated with either supernatants from BYE cultures of wild-type 130b (WT) or *chiA* mutant NU318 (Δ*chiA*). White arrow highlights ChiA-dependent mucin fragment (~95 kDa) and black arrows highlight non-ChiA-dependent mucin fragments (~100 and ~90 kDa). The data presented are representative of three independent experiments. (B) Mucin penetration assay of *L*. *pneumophila* wild-type (WT) and *chiA* mutant (Δ*chiA*) strains applied to the upper chamber of 3.0 μm transwell coated with type II mucin extract. Bacteria that penetrated the transwell were collected from the lower chamber and plated for CFU. Penetration ratio represents CFU in lower chamber 50 or 100 μg mucin / CFU in lower chamber 0 μg mucin. N = 3 experimental replicates. Statistical analysis was done using Two-way ANOVA with Boneferri post-hoc test. Error bars represent standard deviation. *P = <0.05, **P = <0.01. (C) Immunoblot of type II porcine stomach mucin extract incubated with ChiA-FL, subdomains (NTD, CTD), SslE or buffer alone, +/- EDTA, detected with MUC5AC antibody. The bands at ~70 kDa and ~60 kDa correspond to ChiA and SslE processed MUC5AC fragments, respectively.

We next examined whether secreted ChiA is able to degrade mucins. Porcine stomach type II mucin extract was incubated with supernatants from *L*. *pneumophila* 130b wild-type and NU318 (*chiA*) mutant strains or a cocktail of enzymes (pepsin, pronase, β-N-acetylglucosaminidase, fucosidase) with known activity against mucins [39–41], and then analysed by immunoblotting using wheat germ agglutinin (Fig 5A, left panel). While the majority of the mucin extract ran at >500 kDa, after incubation with the mucinase cocktail there was a reduction in high molecular weight species and the appearance of a new band at ~200 kDa. When the extract was incubated with *L*. *pneumophila* 130b wild-type supernatant there was again a reduction of high molecular weight material but with the addition of a new fragment at ~95 kDa. On the other hand, the *chiA* mutant supernatant produced a profile that was more similar to the control, although there was evidence of another new species at ~100 kDa. When the experiment was performed using a greater amount of mucin, the difference between the wild-type and mutant was even more evident (Fig 5A, right panel). Three clear fragments (~100 kDa, ~95 kDa and ~90 kDa) could be observed in the type II mucin extract incubated with wild-type supernatants, with the middle band absent when incubated with the *chiA* mutant supernatant. This band is dependent upon ChiA and implies that ChiA can function as both chitinase and a mucinase. Furthermore, the presence of the ~100 kDa and ~90 kDa fragments in mucin extracts treated with either wild-type or *chiA* mutant supernatants suggests that *L*. *pneumophila* secretes additional mucinase enzymes that are yet to be identified.

Mucins are high molecular weight glycoproteins that contain large numbers of heavily O-glycosylated serine/threonine rich repeat sequences [42]. They exist as cell surface exposed transmembrane proteins or secreted gel-forming proteins of the mucosal barrier and act as a first line of defence against bacterial infection [43]. The normal stomach mucosa is characterised by expression of MUC1, MUC5AC, and MUC6 mucins [44], however, MUC1 and MUC5AC are also major mucins expressed in the mammalian airway and lung [45]. Therefore, to determine whether ChiA can facilitate mucin penetration of *L*. *pneumophila* we performed an artificial mucin penetration assay. After 2 hours incubation with either *L*. *pneumophila* wild-type 130b or *chiA* mutant NU318, we observed a 2.7- and 2.4-fold decrease in the number of colonies from the *chiA* mutant compared with wild-type in the presence of 50 and 100 μg type II mucin extract, respectively (Fig 5B). This confirms that ChiA promotes *L*. *pneumophila* dissemination through mucin layers. We then assessed whether the degradation of mucins by ChiA could also be a source of nutrients for *L*. *pneumophila*. However, *L*. *pneumophila chiA* mutant NU318 grown in chemically defined media, supplemented with up to 100 μg of type II mucin extract, displayed identical growth patterns to the wild-type strain (S8 Fig). This indicates that ChiA-dependent degradation of mucins is not important for *L*. *pneumophila* growth.

### ChiA-CTD is a Zn^2+^-dependent peptidase

We then tested the ability of recombinant ChiA to specifically degrade MUC5AC within type II mucin extracts by immunoblotting and compared its profile to that of recombinant *E*. *coli* SslE. Intact MUC5AC did not enter the gel in the buffer controls and this was likely due to its high carbohydrate content and large mass (>500 kDa before glycosylation). However, incubation of type II mucin extract with ChiA-FL and ChiA-CTD, but not ChiA-NTD, resulted in the processing of MUC5AC into a new ~70 kDa fragment (Fig 5C). When the mucin extract was incubated with SslE, MUC5AC was processed into a different ~60 kDa species. SslE is a member of the M60 family of metalloproteases [38], which use a HExxH motif to coordinates Zn^2+^ in their active site [46]. In the presence of the metal chelating agent ethylenediaminetetraacetic acid (EDTA) we did not detect activity for SslE or ChiA-CTD and this clearly shows that the C-terminal domain of ChiA has dual enzymatic activity. This also suggests that ChiA and SslE use a similar peptidase mechanism for the degradation of mucins.

**Fig 6:**
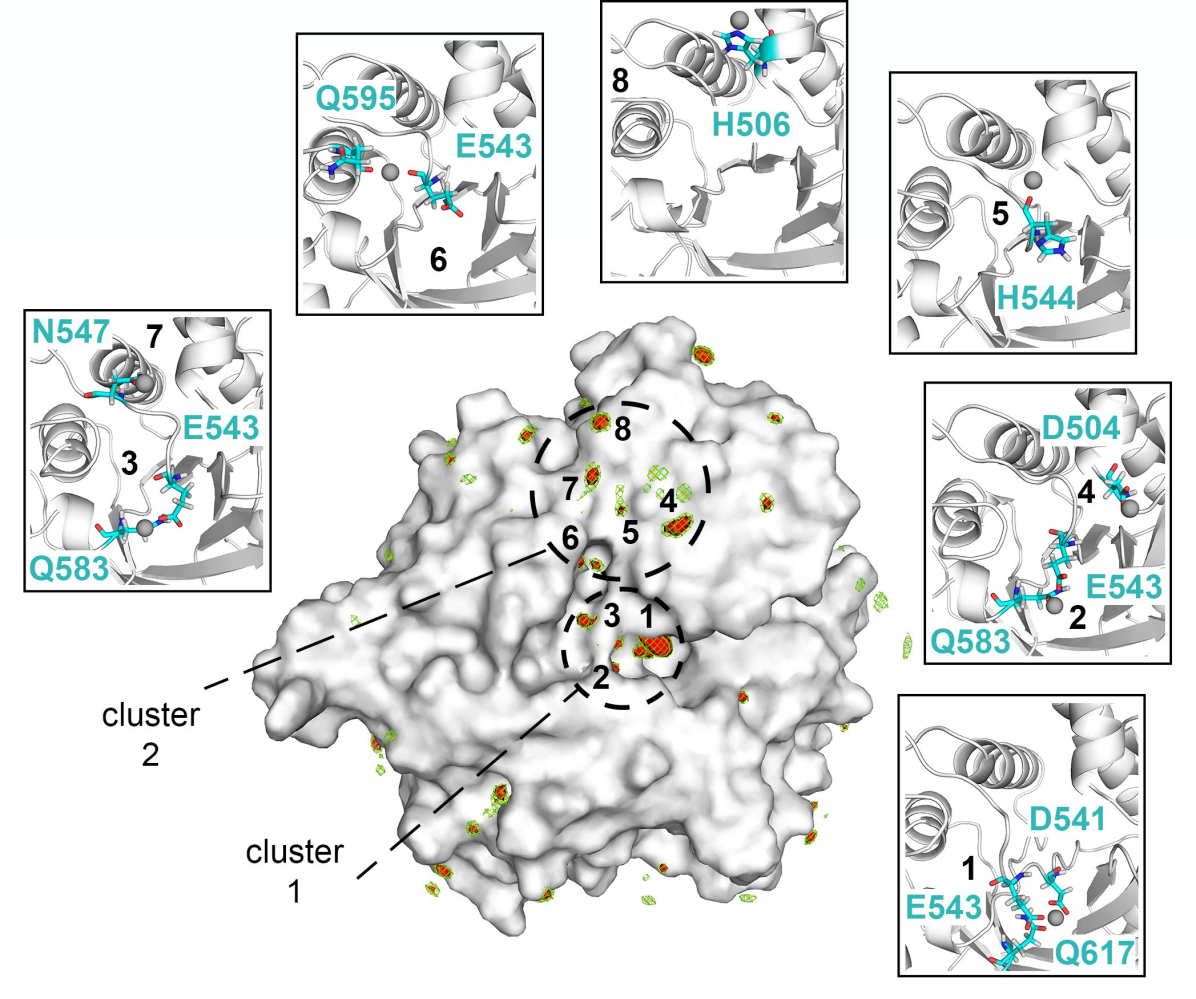
ChiA-CTD Zn^2+^ binding sites. Surface representation of ChiA-CTD showing the spatial distribution of Zn^2+^ ions during MD simulations. The sdf is represented with isosurfaces connecting points with sdf = 20 (green mesh), 25 (yellow mesh) and 30 (red surface) x average sdf. Zn^2+^ high-density sites (red surface) around the chitinase and peptidase active sites are numbered 1 to 8. Blow out boxes show representative structures from the MD simulations to illustrate Zn^2+^ binding in the eight regions, with Zn^2+^ ions shown as spheres, their coordinating residues as sticks and ChiA-CTD as cartoon.

To evaluate this further we performed Molecular Dynamics (MD) simulations and examined the ability of ChiA-CTD to bind Zn^2+^ *in silico*. The protein was ‘soaked’ in a water solution at high Zn^2+^ concentration and the system was then left to evolve over time to identify the regions on the protein surface where Zn^2+^ ions tend to bind. Multiple short simulations were run starting from different random placements of Zn^2+^ ions, for an aggregated simulation time of 1.7 μs. Analysis of the Zn^2+^ spatial distribution function (sdf) calculated on the concatenated trajectories highlighted multiple high Zn^2+^ density sites in the region around the chitinase active site, providing information on the different ways in which Zn^2+^ could bind to the protein in this region. The highest density was found at the chitinase active site (region 1), where Zn^2+^ is coordinated by Asp541, Glu543 and Gln617, with two other sites in close proximity (regions 2,3) coordinated by Glu543 and Gln583 (Fig 6). Binding of two Zn^2+^ions in the active site of *Bacillus cereus* ChiNCTU2 has been shown to inhibit chitinase activity [27] and indicates that metal binding could modulate the different enzyme activities in ChiA. A unique cluster of Zn^2+^ sites was also located away from the chitinase active site, near the MPD-2 ligand site in the ChiA-CTD crystal structure, involving residues Asp504 (region 4), His544 (region 5), Glu543 and Gln595 (region 6), Asn547 (region 7) and His506 (region 8) (Fig 6).

**Fig 7:**
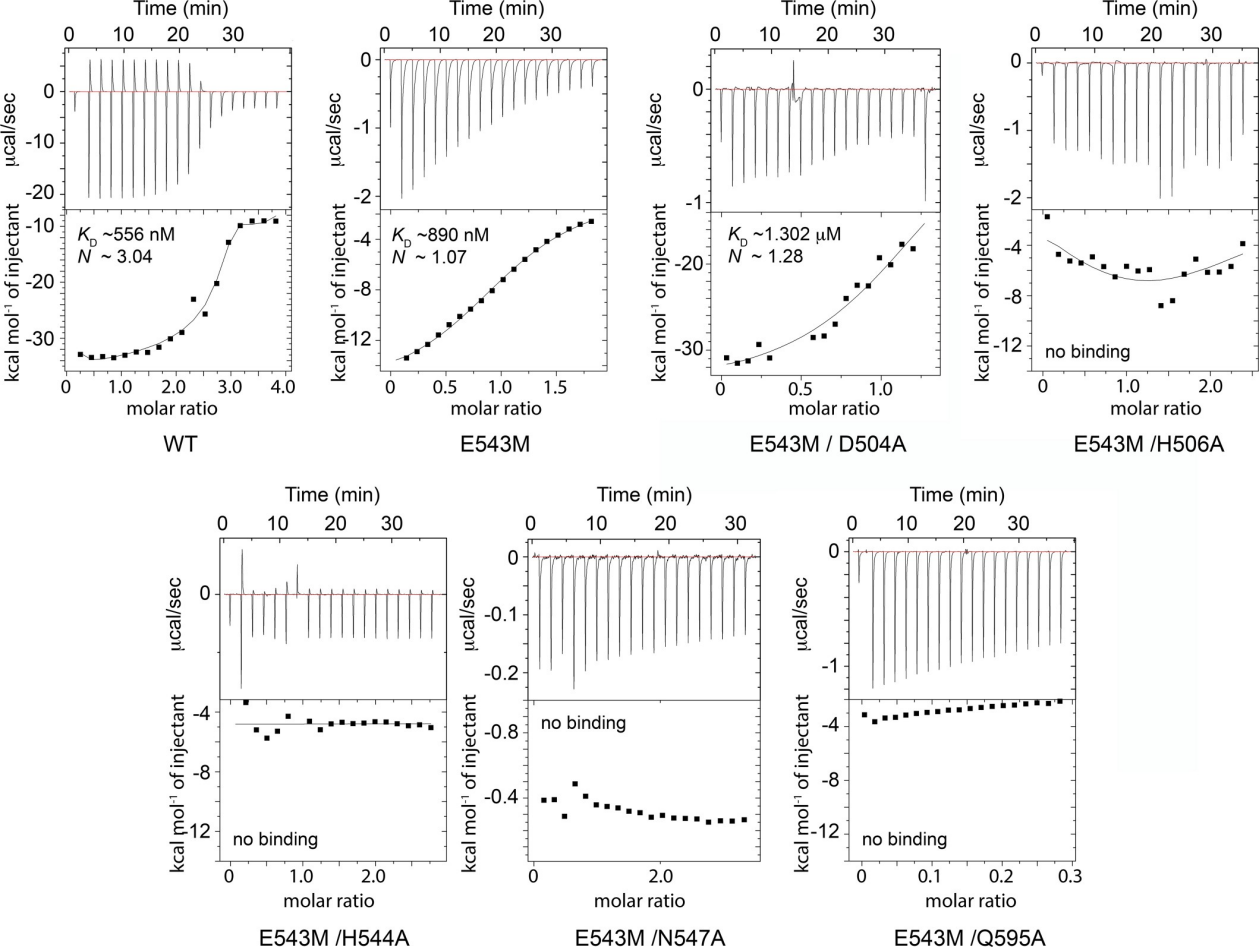
ITC analysis of the interaction of Zn^2+^ with ChiA-CTD. Isothermal titration calorimetry was used to measure the affinities of Zn^2+^ for wild-type ChiA-CTD (WT) and ChiA-CTD variants (E543M, E543M/D504A, E543M/H506A, E543M/H544A, E543M/N547A and E543M/Q595A). Raw data (top) and normalized binding curves (bottom) are reported after subtraction of blank heat pulses. Black squares indicate the normalized heat of interaction obtained per injection, while a black curve represents the best fit obtained by non-linear least-squares procedures based on a 1:1 binding model.

To verify these *in silico* observation we used isothermal titration calorimetry (ITC) and measured an equilibrium dissociation constant (*K*_D_) of 556 nM for approximately three Zn^2+^ ions (*N* = 3.04) binding to wild-type ChiA-CTD (Fig 7). During the experiment we observed both exothermic and endothermic signals, which were not detected in reverse or blank titrations (S9 Fig). This may reflect a different binding mechanism for each site. We then examined binding of Zn^2+^ to ChiA-CTD^E543M^ and observed exothermic binding with a ~1.6-fold reduction in affinity (*K*_D_ 890 nM) at a single site (*N* = 1.07). This indicated that Glu543 is involved in the coordination of zinc in the chitinase active site. To assess whether residues from the second cluster also formed a genuine binding site, we created E543M/D504A, E543M/H506A, E543M/H544A, E543M/N547A and E543M/Q595A double mutants in ChiA-CTD (S2 Fig). When we assessed Zn^2+^ binding to ChiA-CTD^E543M/D504A^ using ITC we measured exothermic binding with a further ~1.5-fold reduction in affinity (*K*_D_ 1.3 μM) at a single site (*N* = 1.28). However, examination of Zn^2+^ binding to ChiA-CTD^E543M/H506A^, ChiA-CTD^E543M/H544A^, ChiA-CTD^E543M/N547A^ and ChiA-CTD^E543M/DQ595A^ showed no binding. This indicates that His506, His544, Asn547 and Q595 form a unique zinc binding site in ChiA.

### ChiA-CTD uses a novel mechanism to cleave mucin-like glycoproteins

**Fig 8:**
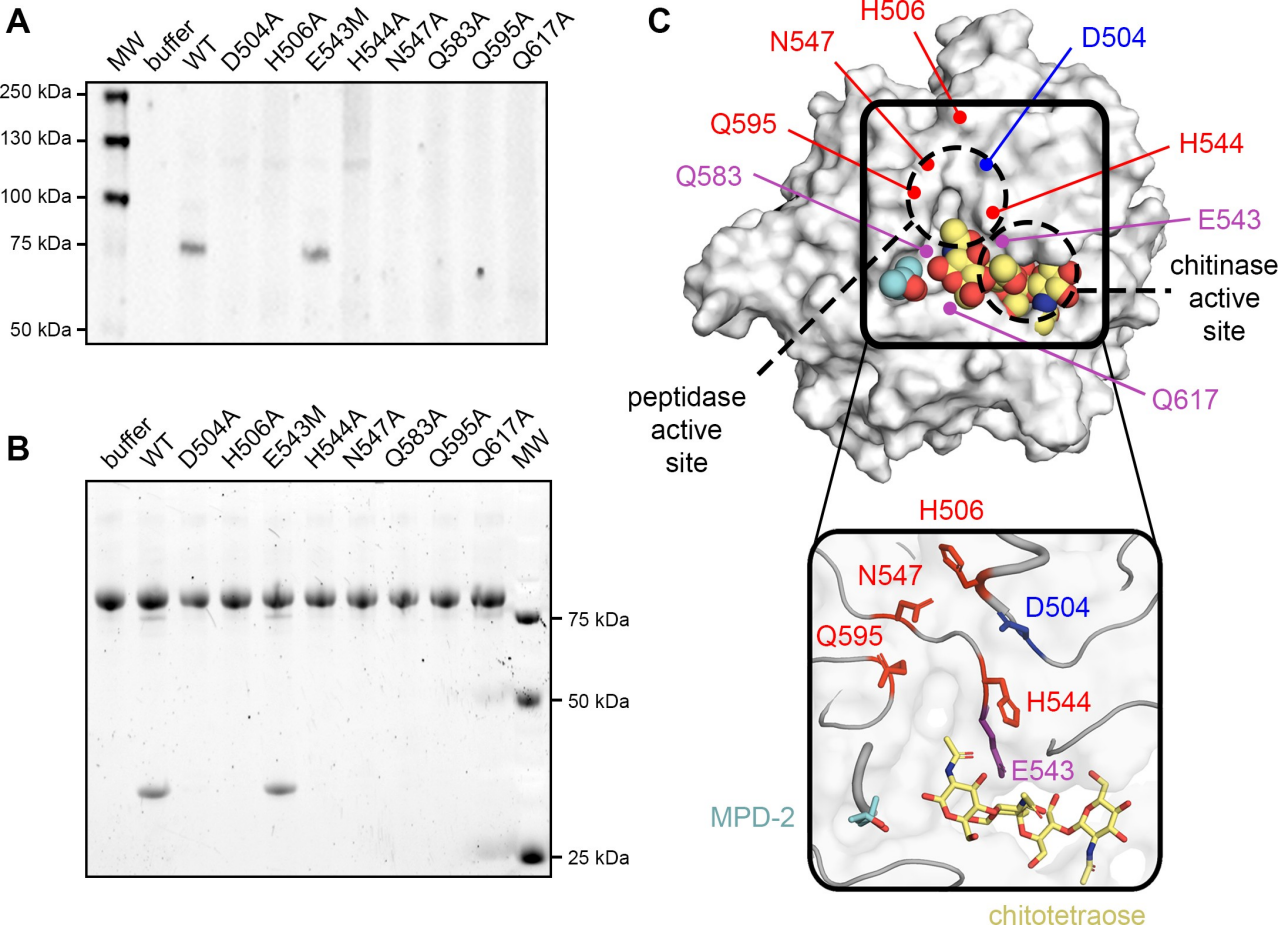
Peptidase active site of ChiA. (A) Immunoblot of type II porcine stomach mucin extract incubated with ChiA-CTD, ChiA-CTD mutants (D504A, H506A, E543M, H544A, N547A, Q583A, Q595A, 617A) or buffer alone and detected with MUC5AC antibody. The band at ~75 kDa corresponds to a ChiA processed MUC5AC fragment. (B) Immunoblot of human C1-INH incubated with ChiA-CTD, ChiA-CTD mutants (D504A, H506A, E543M, H544A, N547A, Q583A, Q595A, 617A) or buffer alone and detected with Pro-Q Emerald 300 glycoprotein stain. The band at ~30 kDa corresponds to a ChiA processed C1-INH fragment. (C) Surface and cartoon representation of ChiA-CTD bound to MPD-2 (cyan; spheres and sticks) and modelled chitotetraose (yellow; spheres and sticks), highlighting the potential for mucin branched glycan recognition. Residues that bind Zn^2+^ or form the metal-dependent aminopeptidase active site are highlighted (purple: chitinase function; red: peptidase Zn^2+^ binding; blue: peptidase general base).

To assess the role of these residues in the degradation of MUC5AC, we created D504A, H506A, H544A, N547A and Q595A single site mutations in ChiA-CTD. All constructs were well folded, retained their ability to bind chitin in pull-down experiments and with our existing single site ChiA variants (ChiA-CTD^E543M^, ChiA-CTD^Q583A^ and CTD^Q617A^), they were still able to bind immobilized type II and III mucin extracts in ELISA assays (S2, S10 and S11 Figs). We then incubated these proteins with type II mucin extract and inspected their MUC5AC degradation profiles by immunoblotting (Fig 8A). Incubation with ChiA-CTD and ChiA-CTD^E543M^ both produced identical MUC5AC degradation patterns, while all other ChiA-CTD variants showed no activity. Human C1-inhibitor (C1-INH) is a serine protease inhibitor with a major role in regulating the contact activation pathway, the classical complement pathway and the lectin complement pathway [47]. C1-INH contains mucin-like glycosylation patterns [48] and is cleaved by other mucin metalloproteases such as enterohemorrhagic *E*. *coli* StcE [49]. When we incubated C1-INH with ChiA-CTD or ChiA-CTD^E543M^ we again measured peptidase activity with a new C1-INH fragment appearing at ~30 kDa. Likewise, in the presence of the other ChiA-CTD mutants we detected no activity (Fig 8B). This demonstrates that ChiA-CTD has broad specificity for mucin-like glycoproteins and its peptidase activity is independent from the adjacent chitinase active site.

## Discussion

Chitin is highly abundant in the environment and can function as a source of carbon and nitrogen [50] but several chitinases have been identified as key virulence factors in bacterial disease [37]. These include *Enterococcus faecalis* efChiA, *E*. *coli* ChiA, *Vibrio cholerae* ChiA2, *Francisella tularensis* ChiA, *Listeria monocytogenes* ChiA and ChiB, *Pseudomonas aeruginosa* ChiC, *Salmonella typhimurium* ChiA and *L*. *pneumophila* ChiA. Although it is unclear how these enzymes perform these dual functions, there is strong evidence that they interact with host glycoconjugates and through their localization and/or enzymatic activity are able to modulate host defence mechanisms [37]. We have determined that *L*. *pneumophila* ChiA has activity against human C1-INH and porcine stomach derived mucins, and the degradation of MUC5AC produces a similar degradation profile to the M60-family *E*. *coli* zinc-aminopeptidase SslE [51, 52]. Recent structural analysis of *Bacteroides thetaiotaomicron* BT4244, *Pseudomonas aeruginosa* IMPa and *Clostridium perfringens* ZmpB M60 proteins has revealed unique structural adaptations that allow them to accommodate different glycan sequences while all cleaving the peptide bond immediately preceding the glycosylated residue [46]. Similarly, *E*. *coli* StcE is an M66-family zinc metalloprotease that recognizes distinct peptide and glycan motifs in mucin-like proteins and then cleaves the peptide backbone using an extended HExxHxxGxxH motif [53, 54]. In StcE, three histidine residues in the conserved motif act as ligands for a single catalytic zinc, while in M60 enzymes two histidines and another residue perform this role [46, 53]. A nucleophilic water molecule is the fourth ligand for the zinc, and this is coordinated by a conserved glutamate, which acts as a general base during catalysis. We have shown that *L*. *pneumophila* ChiA functions in a similar fashion to SslE and StcE but as it does not contain a HExxH motif, ChiA represents a new class of peptidase that can degrade mammalian mucin-like proteins via a novel mechanism.

We have identified four residues in ChiA that are essential for zinc binding in the peptidase active site (His506, His544, Asn547 and Gln595) and these likely coordinate a single zinc ion. These residues along with Asp504, Gln583 and Gln617 are also essential for peptidase activity. Examination of the ChiA-CTD structure suggests that His544, Asn547 and Gln595 are ligands for the zinc, with Asp504 the general base (Fig 8C). Active site zinc ligands are usually histidine, glutamate, aspartate or cysteine residues [55] but neutral residues such as asparagine and glutamine have been observed in other enzymes [56–58]. His506 packs against the N547 and H544 loop and may have a role in correctly structuring this region of ChiA-CTD. However, we cannot rule out the involvement of H544 binding zinc ions in this region. Gln583 and Gln617 have important roles in the optimal positioning of chitin for processing within the chitinase active site (Fig 2B). Lack of peptidase activity in ChiA-CTD^Gln583^ and ChiA-CTD^Gln617^ suggests that these residues are also involved in binding glycan motifs in mucin-like proteins. While Asp504 is located in an augmented α3 helix, His544, Asn547 and Gln595 are positioned within conserved GH18 secondary structure (S4 Fig). However, sequence alignment of *L*. *pneumophila* ChiA with other virulent bacterial chitinases, including the mucin degrading *V*. *cholerae* ChiA2 [59], does not show conservation of these residues and modelling of their tertiary structures using the Phyre2 server [23] also highlights significant differences within their chitin binding surfaces. This implies that other virulent bacterial chitinases either promote pathogenesis using an alternative mechanism or that the specific location of the peptidase active site is unique to each enzyme and shapes their glycan specificity and function.

Mucins derived from the lung are not commercially available, but we have shown that ChiA has specificity for and can degrade MUC5AC purified from the porcine stomach, which is also a major mucin expressed in the human airway and lung [45]. MUC5AC is composed of T-antigen (Galβ1-3GalNAcαSer/Thr), core 2 (GlcNAcβ1-6(Galβ1–3)GalNAcαSer/Thr) and sialyl T-antigen (NeuAcα2-6(Galβ1–3)GalNAcαSer/Thr) core glycan structures [60]. While the T-antigen contains a linear array of carbohydrates, core 2 and sialyl T-antigen have branched structures. Our study suggests that the specificity of ChiA-CTD for O-glycosylated substrates is mediated by glycan recognition in the chitin binding groove, which then orientates the peptide backbone of the substrate towards the peptidase active site for proteolysis (Fig 8C). Furthermore, the MPD-2 site which we observed in the crystal structure of ChiA-CTD may also represent an additional glycan surface to accommodate branched glycan structures.

MUC5AC is a gel forming mucin and our mucin penetration assay indicates that one role for ChiA in the lung is to facilitate bacterial penetration of the alveolar mucosa, which would increase access to host tissue. However, we have also demonstrated that the peptidase activity of ChiA-CTD is active against human C1-INH. This is a major glycoprotein in plasma that regulates the complement pathway through inactivating the proteases C1r, C1s and mannose-binding protease-associated serine protease 2 (MASP2) [61]. In *Bordetella pertussis* the secreted Vag8 protein has been shown to bind and release C1-INH from C1r, C1s and MASP2 [62] where these active proteases can then cleave C2 and C4 from the bacterial surface. This indicates that ChiA may also modulate host immune responses to *L*. *pneumophila* infection via evasion of the complement pathway.

Secreted ChiA has been detected in bacterial culture supernatants and during *L*. *pneumophila* infection of cultured amoebae and human macrophages [17, 32]. We previously observed that in the early stages of macrophage infection ChiA and another type II substrate ProA are exported to the host cytoplasm and localize to the surface of the LCV [32]. The ability of ChiA to bind non-chitin substrates implies that during intracellular infection ChiA interacts with other specific host cytoplasmic glycoproteins and may modulate host pathways either through their requisitioning to the LCV or through their hydrolysis. In this study we have now shown that ChiA can also bind the *L*. *pneumophila* surface, through its N3 domain, although ProA does not. In other bacteria, type II substrates can associate with their outer membrane via acetylation of their N-terminus, interactions with other outer membrane proteins or through recognition of lipopolysaccharides [11, 33]. While the mechanism by which ChiA is targeted to the *Legionella* outer membrane is still unclear, this may be shared during localization of ChiA to the LCV, yet interactions that tether ProA to the LCV are clearly not also present on the *L*. *pneumophila* surface.

Our examination of recombinant ChiA subdomains has revealed that the N-terminal domains and C-terminal chitinase/peptidase domain can all bind porcine stomach mucins and implies that host mucins can be sequestered and processed on the bacterial surface. However, we did not observe significant differences between mucin binding to *L*. *pneumophila* 130b wild-type or *chiA* mutant strains and this demonstrates that additional uncharacterized mucin binding proteins may be present on the bacterial surface. We also identified additional mucinase activity in the culture supernatants of both *L*. *pneumophila* 130b wild-type and *chiA* mutant strains, which implies that other yet to be identified mucinase enzymes are secreted by *L*. *pneumophila*, compatible with past bioinformatic assessments of the *L*. *pneumophila* genome [10]. Together this indicates that manipulation of the host mucosa is an important pathogenic mechanism of *L*. *pneumophila*. The multifaceted nature of ChiA makes it a highly versatile virulence factor of *L*. *pneumophila* and likewise a target for controlling *L*. *pneumophila* infection. As a surface associated protein ChiA is a promising vaccine target and our structural characterization may provide a platform to initiate vaccine development.

## Materials and methods

### Cloning, expression and purification

Full-length ChiA (ChiA-FL; residues 1–762), minus the N-terminal periplasmic signal sequence, the ChiA N-terminal region (ChiA-NT; residues 1–417), the ChiA N1-domain (ChiA-N1; residues 1–140), the ChiA N2-domain (ChiA-N2; residues 138–299), the ChiA N3-domain (ChiA-N3; residues 285–417) the ChiA C-domain (ChiA-CTD; residues 419–762) and NttE (residues 1–269) were amplified from the genomic DNA of *L*. *pneumophila* strain 130b and cloned into the N-terminal His_6_-tagged vector pET-46 Ek/LIC (S4 Table). SslE (residues 67–1497), minus the N-terminal periplasmic signal sequence and mature SslE N-terminal proline-rich region, were amplified from the genomic DNA of *E*. *coli* strain W and cloned into the C-terminal His_6_-tagged vector pOPINE (S4 Table). These were transformed into *E*. *coli* SHuffle cells (New England Biolabs) and grown at 37°C in LB media with 100 μg/ml ampicillin. Expression was induced with 1 mM isopropyl-d-1-thiogalactopyranoside (IPTG) at an OD_600nm_ of 0.6 and cells were harvested after growth overnight at 18°C. Cells were resuspended in 20 mM Tris–HCl pH 8, 200 mM NaCl, 5 mM MgCl_2_, 1 mg/ml DNase I, 5 mg/ml lysozyme, lysed by sonication and purified using nickel affinity chromatography. All samples were then gel filtered using a Superdex 200 column (GE Healthcare) equilibrated in 20 mM Tris–HCl pH 8, 200 mM NaCl.

### Nuclear magnetic resonance spectroscopy

One-dimensional proton NMR experiments were performed at 25°C on 300 μM ChiA-NT, ChiA-N1, ChiA-N2, ChiA-N3 and ChiA-CTD samples in a buffer containing 20 mM Tris-HCl pH 8.0, 100 mM NaCl, 10% D_2_O. Spectra were recorded on a Bruker Avance III 700 MHz (ChiA-N1, ChiA-N2, ChiA-N3, ChiA-CTD) or 800 MHz (ChiA-NT) spectrometer equipped with cryoprobes and processed within TopSpin (Bruker).

### Site-directed mutagenesis

E543M *chiA-CTD* mutant was created using pET46*chiA-CTD* template DNA with a QuikChange II Site-Directed Mutagenesis Kit (Stratagene) (S4 Table). D504A, H506A, H544A, N547A, Q583A, Q595A, Q617A, E543M/D504A, E543M/H506A, E543M/H544A, E543M/N547A and E543M/Q595A *chiA-CTD* mutants were synthesized by Synbio Technologies and cloned into pET28b vector using NcoI and XhoI restriction sites (S5 Table). All resulting clones were verified by DNA sequencing and then expressed and purified as described for wild-type ChiA-CTD.

### Circular dichroism

Far-UV CD spectra were measured in a Chirascan (Applied Photophysics) spectropolarimeter thermostated at 20°C. Spectra for wild-type ChiA-CTD and ChiA-CTD^D504A^, ChiA-CTD^H506A^, ChiA-CTD^E543M^, ChiA-CTD^H544A^, ChiA-CTD^N547A^, ChiA-CTD^Q583A^, ChiA-CTD^Q595A^, ChiA-CTD^Q617A^, ChiA-CTD^E543M/D504A^, ChiA-CTD^E543M/H506A^, ChiA-CTD^E543M/H544A^, ChiA-CTD^E543M/N547A^ and ChiA-CTD^E543M/Q595A^ mutants (0.05 mg/ml) in 10 mM Tris-HCl pH 8.0, 100 mM NaCl were recorded from 260 to 200 nm, at 0.5 nm intervals, 1 nm bandwidth, and a scan speed of 10 nm/min. Three accumulations were averaged for each spectrum.

### Chitinase activity assay

Enzyme activity was determined using 4-Nitrophenol β-D-N,N’,N”-triacetylchitotriose (Sigma) as a substrate. All experiments were performed in triplicate. 10 μl of ChiA-FL, ChiA-NT, ChiA-CTD, ChiA-CTD^E543M^, ChiA-CTD^Q583A^ or ChiA-CTD^Q617A^ at 10 μg/ml in PBS were mixed with 90 μl of substrate at 0.4 mg/ml dissolved in 20 mM sodium acetate pH 4.8. These samples and a 300 μl standard (50 μM ρ-nitrophenol, 100 mM sodium carbonate) were incubated at 37°C for 30 min and then the ChiA reactions were quenched with the addition of 200 μl of 100 mM sodium carbonate. The release of the chromophore ρ-nitrophenol (pNP) was measured at 405 nm and ChiA samples were corrected for absorption in a control sample with added PBS instead of protein. 1 unit of activity per mg enzyme (U/mg) was defined as the release of 1 mmol of pNP/mg of ChiA/min.

### Chitin binding assay

250 μl ChiA-FL, ChiA-NT, ChiA-N1, ChiA-N2, ChiA-N3, ChiA-CTD, ChiA-CTD^D504A^, ChiA-CTD^H506A^, ChiA-CTD^E543M^, ChiA-CTD^H544A^, ChiA-CTD^N547A^, ChiA-CTD^Q595A^ and BSA (Sigma) at 10 μM in 20 mM Tris-HCl pH 8.0, 200 mM NaCl were incubated with 50 μl chitin-resin (Sigma) and incubated whilst shaking for 30 min. The resin was washed three times with 500 μl of the same buffer and then proteins were eluted by incubating the resin in 250 μl of 8 M urea, 1% (w/v) SDS for 30 min whilst shaking. Protein samples prior to incubation with chitin-resin and the eluted protein/chitin-resin slurry were then analysed with SDS-PAGE. Eluted samples underwent an upward shift compared to the input samples due to the large differences in buffer conditions.

### Crystal structure determination

Crystallization of ChiA CTD-domain (30 mg/ml) was performed using the sitting-drop vapour-diffusion method grown in 0.2 M ammonium acetate, 0.1 M Bis-Tris pH 5.5, 45% (v/v) 2-Methyl-2,4-pentanediol at 293K. Native crystals were flash cooled in liquid nitrogen and diffraction data were collected at 100K on beamline I04 at the Diamond Light Source (DLS), UK. Crystals were also soaked for 1 min in well solution containing 1.0 M NaI, flash cooled in liquid nitrogen and data were collected at 100K on beamline I02 at the Diamond Light Source (DLS), UK. Data were processed with XDS [63] and scaled with AIMLESS [64] using the XIA2 pipeline [65]. The structure of ChiA CTD-domain was determined with I-SIRAS. Twenty-one iodide sites were located in ChiA C-domain using SHELXD [66], and then phases were calculated using autoSHARP [67]. After automated model building with ARP/wARP [68], the remaining structure was manually built within Coot [69]. Refinement was carried out with REFMAC [70] using non-crystallographic symmetry (NCS) and translation-libration-screw (TLS) groups [71, 72], and 5% of the reflections were omitted for cross-validation. Processing and refinement statistics of the final model can be found in Table 1.

### SAXS data collection and analysis

SAXS data were collected on beamline B21 at the Diamond Light Source (DLS), UK at 20°C. Full-length ChiA in 20 mM Tris–HCl pH 8, 200 mM NaCl were measured at 4, 2, 1 and 0.5 mg/ml concentrations, after gel filtration using a Superdex 200 column (GE Healthcare), over a momentum transfer range of 0.004<*q*<0.4 Å^−1^. A fresh sample of BSA was measured as a standard. Buffer subtraction, intensity normalization, and data merging for the different sample concentrations were performed in SCATTER (DLS, UK). ChiA data collected above 1 mg/ml showed signs of aggregation and were discarded. Further analysis was carried out with the 1 mg/ml data using a q range 0.008<*q*<0.2 Å^−1^. The radius of gyration (Rg) and scattering at zero angle (I(0)) were calculated from the analysis of the Guinier region by AUTORG [73, 74]. The distance distribution function (P(r)) was subsequently obtained using GNOM [73, 74], yielding the maximum particle dimension (D_max_). Determination of molecular model ensembles that best fit the SAXS data was performed using EOM2.0 [29, 30]. An initial model of ChiA was created from PHYRE2 models of the N1- and N3-domains (residues 7–132; 300–399), a ROBETTA model of the N2-domain (residues 138–290) and our crystal structure of the C-domain (residues 424–762), with domain linker sequences kept unstructured. SAXS structural and EOM parameters can be found in S2 Table and S3 Table, respectively.

### ELISA for detection of ChiA on bacterial surface

Bacterial whole-cell ELISA was done as previously described [75], with slight modification. Wild-type *L*. *pneumophila* strain 130b and isogenic mutants lacking either *chiA* (strain NU318) [17], *mip* (strain NU203) [35], or *proA* (strain AA200) [76] were grown on BCYE agar for 3 days at 37°C. Using a sterile cotton swab, bacteria were resuspended in 1 ml sterile PBS to an OD_660_ 0.3, centrifuged at 10,000 x *g* for 3 min, and then washed once with PBS to remove debris and unbound proteins. Bacteria were fixed in 4% (w/v) paraformaldehyde for 10 min, followed by two 1-ml washes in PBS. Bacteria were resuspended in coating buffer (100 mM bicarbonate/carbonate buffer, pH 9.6) to a final OD_660_ 0.03, and 100 μl of this suspension were added into the wells of Nunc MaxiSorp immunoassay plates (Thermo Fisher Scientific). Following overnight incubation at 4°C, the wells were washed three times with 200 μl of wash buffer (PBS + 0.05% Tween-20), and then 200 μl of blocking buffer (PBS + 0.05% Tween-20 + 5% dried milk) were added for 1 h at 25°C. After removal of the blocking buffer, samples were incubated with 100 μl of primary antibody (i.e., rabbit anti-Mip [34], rabbit anti-ChiA [32], or rabbit anti-ProA [32] diluted 1:10,000 in blocking buffer for 1 h at 25°C. Following three, 200-μl washes with wash buffer, samples were incubated with 100 μl of secondary antibody (anti-rabbit conjugated HRP) diluted 1:1,000 in blocking buffer for 1 h at 25°C. Following five washes with 200 μl wash buffer, samples were incubated with 100 μl 3,3',5,5'-Tetramethylbenzidine (TMB) substrate for 15 min at 25°C, and then, the reaction was stopped by addition of 50 μl of 2 N sulfuric acid. Absorbance values were measured at 450 nm with wavelength correction of 570 nm using a microplate reader (Synergy H1, BioTek). To confirm that bacterial lysis had not occurred during sample processing and plate coating, *L*. *pneumophila*-coated wells were probed with an ICDH-specific antiserum that recognizes a cytosolic *L*. *pneumophila* protein [77], and no signal was detected as compared to wells coated with *L*. *pneumophila* lysed by freeze-thaw lysed (S12 Fig). In order to assess the binding of recombinant ChiA and ChiA subdomains to the *L*. *pneumophila* surface, *chiA* mutant NU318 was grown on BCYE agar, washed, and resuspended in PBS, as indicated above. Prior to fixation, bacteria resuspended to OD_660_ 0.3 were incubated with 1 μg of recombinant protein in the presence of 1x protease inhibitors (Pierce, Thermo Scientific) in sterile 1.5 ml microcentrifuge tubes with gentle end-over-end mixing for 30 min at 25°C. Following two 1-ml washes in PBS to remove unbound protein, bacteria were fixed with 4% (w/v) paraformaldehyde and processed for ELISA as described above. Preliminary ELISA assays determined that the polyclonal anti-ChiA [32] antiserum was capable of recognizing each of the recombinant ChiA fragments when they were added to wells in the absence of bacteria. To that end, 10 ng of each protein within 100-μl of coating buffer was added to wells, and then allowed to adhere overnight at 4°C, before exposure to the antibodies (diluted 1:10,000) and ELISA, as described above. Wells coated with coating buffer only were used as background controls.

### Mucin binding ELISA

Immulon 2-HB 96-well plates (VWR) were coated overnight at 4°C with 50 μl of partially purified mucins from bovine submaxillary glands (type I-S; Sigma) and porcine stomachs (type II and III; Sigma) at 100 μg/ml in 50 mM Carbonate/Bicarbonate pH 9.6. Wells were blocked for 1 hr at 25°C with 200 μl of 0.1% (w/v) bovine serum albumin (BSA) in PBS–0.05% Tween 20 and then washed once with 200 μl of incubation buffer (0.05% (w/v) BSA in PBS–0.05% Tween 20). Wells were then incubated for 3 hrs at 25°C with 50 μl of ChiA-FL, ChiA-NT, ChiA-N1, ChiA-N2, ChiA-N3, ChiA-CTD, ChiA-CTD^D504A^, ChiA-CTD^H506A^, ChiA-CTD^E543M^, ChiA-CTD^H544A^, ChiA-CTD^N547A^, ChiA-CTD^Q583A^, ChiA-CTD^Q595A^, ChiA-CTD^Q617A^, NttE and SslE at 10 μM in incubation buffer. This was followed by four washes with 200 μl of incubation buffer and incubation with 50 μl of anti-His-HRP antibody (Sigma), diluted 1:2000 in incubation buffer for 1 hr at 24°C. After four washes with 200 μl of incubation buffer, 150 μl of *o*-Phenylenediamine dihydrochloride (Sigma) was added for 30 min and then data was recorded at 450 nm.

### Assay for mucin binding to bacteria

*L*. *pneumophila* wild-type strain 130b and *chiA* mutant NU319, both harbouring a GFP-expressing plasmid [17, 32], were incubated for 3 days on BCYE agar containing IPTG at 1mM. Bacteria were suspended in PBS to an OD of 0.3 (i.e., 1 x 10^9^ CFU per ml), and then 1 ml of the suspension was statically incubated with either 100 μg of type II porcine stomach mucins or type III porcine stomach mucins or PBS alone for 1 h at 25°C or 37°C. Following three washes each consisting of a 1-ml PBS wash solution and a 5-min centrifugation step at 4000 x *g* (for the 37°C samples) or 8000 x *g* (for the 25°C samples), bacteria were incubated with 7.5 μg of Texas Red-tagged wheat germ agglutinin (WGA) for 15 min at 25°C in order to detect mucins bound to the bacteria. After three further washes, as indicated above, the bacteria were resuspended in 1 ml PBS and finally analysed on a BD LSRII flow cytometer using a Texas Red filter and GFP Filter [78].

### Immunoblot for detecting secreted mucinase activity

*L*. *pneumophila* strains that had been grown for three days on BCYE agar were suspended into 20 ml of BYE broth to an OD_660_ of 0.3 and grown overnight at 37°C to an OD_660_ of 3.0–3.3. Bacteria were sub-cultured into fresh BYE medium to an OD_660_ = 0.3 and grown, with shaking, to an OD_660_ of 1.0, which corresponded to the mid-log phase. Supernatants were collected, filtered through a 0.22-μm filter, and concentrated using 10-kDa Amicon concentrators (EMD Millipore). 200 μl of concentrated supernatants were incubated with 200 or 400 μg of type II porcine stomach mucins. As controls, the mucins were either incubated in uninoculated BYE broth or in BYE broth containing 50 μl of a known mucinase cocktail, which consisted of 10 μl each of pepsin (0.5 mg/ml), pronase (10 mg/ml), β-N-acetylglucosaminidase (2.5 μM), fucosidase (5 U/ml), and DTT (1 mM) dissolved in 940 μl of ddH_2_0. The various samples were incubated statically for 3 h at 25°C and then subjected to electrophoresis prior to immunoblotting [79]. Reactions were stopped by adding 200 μl of 2x Laemmli buffer and incubating for 5 min at 100°C, and 35 μl of each sample was electrophoresed through a Criterion 4–20% SDS-PAGE gel (Bio-Rad) for 1.5 h at 250 volts. The separated reaction products were transferred onto PVDF membrane over the course of 13 min using the semi-dry Invitrogen Power-Blotter and Power Blotter transfer blotting solution. Following incubation in 1% BSA in TBST for 1 h at room temperature, the membranes were incubated overnight at 4°C with biotinylated wheat germ agglutinin that had been diluted 1:2000 (from a 1 mg/ml stock) in TBST with BSA. After three, 5-min washes with TBST buffer, the membranes were further incubated for 1 h at 37°C with Avidin-HRP that had been diluted 1:2000 in BSA-containing TBST. Finally, subsequent to a series of washes, the blot was incubated for 1 min in 2 ml Amersham ECL reagent and then exposed to X-ray film.

### Mucin coated transwell penetration assay

*L*. *pneumophila* wild-type 130b (WT) and *chiA* mutant NU318 (*chiA*) were grown for three days on BCYE agar and then resuspended into 20 ml of BYE broth to an OD_660_ of 0.3 and grown overnight at 37°C to an OD_660_ of 3.0–3.3. Bacteria were sub-cultured into fresh BYE medium to an OD_660_ of 0.3 and grown, with shaking, to an OD_660_ of 1.0, which corresponded to the mid-log phase. Bacteria were then diluted in BYE broth to OD_660_ of 0.3. Transwell plates (Corning) were used for analysis of bacterial crossing of a mucin layer following a previously described protocol [80, 81]. 500 μl of sterile BYE broth was added to the bottom of either empty wells, or wells containing 3.0 μm transwells. Transwells were either kept uncoated or coated with 50 or 100 μg of type II porcine mucin in bicarbonate buffer. After 1 hr of coating transwells with either control BYE broth or type II mucin, 500 μl of 0.3 OD_660_ *L*. *pneumophila* (*chiA or* WT) was applied to either the empty well, or to the top of a transwell. Bacteria that were able to cross the transwell membrane to the bottom of the well were collected 2 hrs after application to wells, diluted and plated onto BCYE plates for CFU analysis. Each experiment had 3 technical replicates. N = 3 experimental replicates were analysed. Two-way ANOVA with Boneferri post-hoc statistical analysis was used.

### *L*. *pneumophila* growth on CDM with mucin

*L*. *pneumophila* wild-type 130b (WT) and *chiA* mutant NU318 (*chiA*), that had been grown for three days on BCYE agar were suspended into 20 ml of BYE broth to an OD_660_ of 0.3 and grown overnight at 37°C to an OD_660_ of 3.0–3.3. Bacteria were washed three times and then subcultured to an OD_660_ of 0.3 in fresh chemically defined medium (CDM), as previously described [82–84]. Bacteria were then incubated at 37°C, with shaking, in CDM containing either 0 μg/ml, 50 μg/ml, or 100 μg/ml porcine mucin II. Growth was assessed by plating aliquots of the cultures on BCYE agar [85, 86] at 0 h, 8 h and 24 h.

### Immunoblot for detecting recombinant ChiA MUC5AC activity

Porcine type II stomach mucin (Sigma) was dissolved in PBS at 8 mg/ml and incubated for 5 min with 5 mM EDTA to remove divalent cations. This was then buffer exchanged into PBS using a 30–50 kDa MWCO concentrator (Generon). Recombinant ChiA-FL, ChiA-NT, ChiA-CTD, ChiA-CTD^D504A^, ChiA-CTD^H506A^, ChiA-CTD^E543M^, ChiA-CTD^H544A^, ChiA-CTD^N547A^, ChiA-CTD^Q583A^, ChiA-CTD^Q595A^, ChiA-CTD^Q617A^ and SslE were incubated for 5 min with 5 mM EDTA to remove any bound metal ions. These were then dialyzed extensively against PBS with 1 mM ZnCl_2_ and the concentrations adjusted to 20 μM. Mucins were mixed with an equal volume of protein in either PBS, 1 mM ZnCl_2_ or PBS, 5 mM EDTA and incubated for 3 hr at 25°C. Reactions were stopped with the addition of an equal volume of 2x Laemmli buffer and incubated for 5 min at 100°C. Samples were then run on a Criterion 4–20% SDS-PAGE gel (Bio-Rad), followed by transfer onto a PVDF membrane using the semi-dry Invitrogen Power-Blotter and Power Blotter transfer blotting solution. The membrane was incubated in 1% BSA in TBST for 1 h at room temperature, and then overnight at 4°C with biotin conjugated MUC5AC antibody (Thermo Fisher Scientific) that had been diluted 1:2000 in TBST with BSA. After three, 5-min washes with TBST buffer, membranes were incubated for 1 h at 37°C with Avidin-HRP diluted 1:2000 in BSA-containing TBST and followed by three washes for 5-min each. This was then incubated with avidin-HRP (1:2000 dilution) for 1 hr at 25°C and then treated with enhanced chemiluminescence substrate (ECL; Pierce) before detection by enhanced chemiluminescence.

### Immunoblot for detecting recombinant ChiA C1-INH activity

Recombinant ChiA-CTD, ChiA-CTD^D504A^, ChiA-CTD^H506A^, ChiA-CTD^E543M^, ChiA-CTD^H544A^, ChiA-CTD^N547A^, ChiA-CTD^Q583A^, ChiA-CTD^Q595A^ and ChiA-CTD^Q617A^ were incubated for 5 min with 5 mM EDTA to remove any bound metal ions. They were then dialyzed extensively against PBS with 1 mM ZnCl_2_ and the concentrations adjusted to 50 μg/ml. 50 μl of ChiA sample was mixed with 50 μl of human C1-INH (1 mg/ml; Sigma) and incubated for 3 hr at 25°C. Reactions were stopped by adding 10 μl of 0.5 M EDTA. Samples were then run on a Criterion 4–20% SDS-PAGE gel (Bio-Rad) and visualized using Pro-Q Emerald 300 glycoprotein stain (Thermo Fisher Scientific).

### Molecular Dynamics

MD simulations and analyses were performed using GROMACS 2016 v3 [87] using a protocol similar to Ref [88]. The protein was described using the Amber99SB*-ILDN force field [89] and solvated using a truncated octahedral box of TIP3P water molecules. A minimal distance of 12 Å was set between the protein and the walls of the box. The charge of the ionisable residues was set to that of their standard protonation state at pH 7. Zn^2+^ ions were added by randomly replacing water molecules. A high Zn^2+^concentration (0.75 M) was used to have a faster sampling of possible Zn^2+^ sites around the protein surface. Cl^-^ counterions were added to neutralise the system.

Periodic boundary conditions were applied. The equations of motion were integrated using the leap-frog method with a 2-fs time step. The LINCS [90] algorithm was chosen to constrain all covalent bonds in the protein, while SETTLE [91] was used for water molecules. The Particle Mesh Ewald (PME) [92] method was used for electrostatic interactions, with a 9-Å cut-off for the direct space sums, a 1.2-Å FFT grid spacing, and a 4-order interpolation polynomial for the reciprocal space sums. A 9-Å cut-off was used for van der Waals interactions. Long-range corrections to the dispersion energy were included.

Each system was minimised through 3 stages with 2000 (positional restraints on heavy atoms) + 3000 steps of steepest descent, followed by 2000 steps of conjugate gradient. Positional restraints on heavy atoms were initially set to 4.8 kcal/mol/Å^2^ and they were gradually decreased to 0 in 1.5 ns, while the temperature was increased from 200 to 300 K at constant volume. The system was then allowed to move freely and was subjected to 1-ns equilibration in NVT conditions at T = 300 K. This was followed by a 2-ns equilibration in NPT conditions with T = 300 K and p = 1 bar. For these equilibration steps, the Berendsen [93] algorithm was used for both temperature and pressure regulation with coupling constants of 0.2 and 1 ps, respectively. At last, a 2-ns NPT equilibration was run after switching to the v-rescale thermostat [94] with a coupling constant of 0.1 ps and the Parrinello-Rahman barostat [95] with a coupling constant of 2 ps. Production NPT runs were then performed for 50 ns, saving the coordinates every 1 ps. Multiple replicas (34) were run, with each replica starting from a different configuration of the ions around the protein, for an aggregated simulation time of 1.7 μs (34 X 50 ns).

The spatial distribution function [96] (sdf) of Zn^2+^ around the protein was calculated with the *gmx spatial* tool from GROMACS. Trajectories from the different replicas were first concatenated together and each frame was aligned through a best-fit superposition to the starting frame using the protein coordinates. A 0.5-Å grid spacing was used for the sdf calculation. The average of non-null sdf values was calculated and isosurfaces connecting points with sdf = 20, 25 and 30 x average sdf were considered.

### Isothermal calorimetry

ITC experiments were performed at 293 K using a MicroCal iTC200 calorimeter (Malvern). ChiA-CTD, ChiA-CTD^E543M^ ChiA-CTD^E543M/D504A^, ChiA-CTD^E543M/H506A^, ChiA-CTD^E543M/H544A^, ChiA-CTD^N547A^ and ChiA-CTD^Q595A^ were dialyzed into buffer containing 20 mM Tris pH 8.0, 200 mM NaCl. Experiments were performed by placing the solution containing ChiA proteins in the cell at 70 μM and the solution containing the zinc (dissolved in dialysis buffer) in the syringe at 2 mM. For each titration 18 injections of 2 μl were performed. Integrated data, corrected for heats of dilution, were fitted using a nonlinear least-squares algorithm to a 1:1 binding curve, using the MicroCal Origin 7.0 software package. Each experiment was repeated at least twice, and representative values are reported.

## Supporting information

S1 Fig 1D 1H NMR spectra of ChiA subdomains. The methyl region of the NMR spectra includes high-field proton resonances observed at low chemical shifts (<0.5 ppm), which indicate the presence of characteristic clusters of aromatic and methyl groups in the core of a structured protein. In addition, the envelope of peaks resonating at high chemical shift (>8.5 ppm) correspond to highly ordered backbone amides present in secondary structure elements.
(TIF)

S2 Fig Circular dichroism (CD) spectra of ChiA-CTD constructs. The negative bands between ~210 to ~ 220 nm and positive band at 200 nm is indicative of a mixed α/β protein fold. The spectra for wild-type (WT) ChiA-CTD and mutants are in essence identical and demonstrates that these mutations do not perturb the structure of the CTD domain.
(TIF)

S3 Fig MPD bound to ChiA-CTD. Electrostatic surface potential representation of ChiA-CTD with two molecules of MPD shown as spheres (MPD1: 4S enantiomer; MPD2: 4R enantiomer). Each binding site is expanded and the ρA weighted electron density maps contoured at 1.0 r.m.s. are shown.
(TIF)

S4 Fig Sequence alignment of *Legionella pneumophila* ChiA-CTD and *Bacillus cereus* ChiNCTU2. Secondary structure elements of ChiNCTU2 and ChiA-CTD are shown above and below, respectively (green rectangle: α-helix; gold arrow: β-strand). Amino acid identities and similar residues are indicated by background shading in cyan and yellow, respectively. Catalytic chitinase residues and chitin binding residues in ChiNCTU2 are indicated with red and blue filled circles, respectively. Mucinase active site residues in ChiA-CTD are shown as open red circles.
(TIF)

S5 Fig Superposition of ChiA-CTD tertiary homologs. *L*. *pneumophila* ChiA-CTD is green, *Bacillus cereus* ChiNCTU2 is purple (PDB ID code 3n18) [27], *Bacillus anthracis* Chi36 is red (PDB ID code 5kz6, *Chromobacterium violaceum* ChiA is yellow (PDB ID code 4tx8) and *Streptomyces coelicolor* ChiA is blue (PDB ID code 3ebv). Augmented loop and helical structures in *L*. *pneumophila* ChiA-CTD are annotated.
(TIF)

S6 Fig SAXS analysis of ChiA-FL. (A) Comparison of scaled scattering curves of ChiA-FL at 0.5 mg/ml (black), 1.0 mg/ml (red) and 2.0 mg/ml (teal) to highlight aggregation at concentrations above 1.0 mg/ml. (B) Experimental scattering curve of ChiA-FL (black open circles). Inset: Guinier Region (orange open circles) and linear regression (black line) for Rg evaluation. (C) Shape distribution [P(r)] function derived from SAXS analysis for ChiA. (D) Kratky, (E) Kratky-Debye and (F) Porod-Debye plots indicate that ChiA is a highly dynamic particle in solution.
(TIF)

S7 Fig Antibody binding to recombinant ChiA fragments. ELISA analysis of anti-ChiA antibodies binding to either full-length ChiA (FL), the N-terminal domain of ChiA (NT), and the C-terminal domain of ChiA (CTD) (left panel) or the ChiA N-terminal subdomain 1 (N1), subdomain 2 (N2), and subdomain 3 (N3) (right panel). All values represent the mean and standard deviation from triplicate wells.
(TIF)

S8 Fig *L*. *pneumophila* growth on mucin supplemented media. WT and *chiA* mutant bacteria were grown from a starting OD_660_ of 0.3 in chemically defined medium in the presence of porcine mucin II at the indicated concentrations. At 0 h, 8 h and 24 h, bacterial numbers were determined by plating for CFU. N = 3. Representative graph shown above as mean and standard deviation of technical replicates in triplicate. Two other experiments showed the same trends, with no significant difference between mutant or mucin effect.
(TIF)

S9 Fig Reverse ITC titration. Titration of ChiA-CTD (syringe) into Zn^2+^ (cell) to assess heat generation during the dilution of ChiA-CTD. No significant heat generation was observed.
(TIF)

S10 Fig Chitin-resin pull down with ChiA mutants. SDS-PAGE gels loaded with ChiA-CTD mutants or BSA control either before incubation with chitin beads (L) or after elution from the beads (B). Eluted samples undergo an upward shift compared to the input sample due to differences in buffer conditions. Data is representative of three independent repeat experiments.
(TIF)

S11 Fig Mucin binding of ChiA-CTD mutants. ELISA analysis of binding between immobilised type II or III mucin extracts and His-tagged wild-type ChiA-CTD (WT), ChiA-CTD mutants (D504A, H506A, E543M, H544A, N547A, Q583A, Q595A, 617A) and controls (SslE, NttE). Anti-His-tag antibody conjugated to HRP was used to measure OD_450 nm_ values. BSA-coated wells were used as controls. Data represent the mean and standard deviation for triplicate experiments. *, *P* < 0.001; verses control empty well by two-tailed Student’s test.
(TIF)

S12 Fig Controls for detection of proteins bound to *L*. *pneumophila* surface. Whole cell ELISA of *L*. *pneumophila* wild-type 130b (WT) and *mip* mutant NU203 (*mip*) detected with Mip-specific antiserum, and *L*. *pneumophila* wild-type 130b (WT) and *L*. *pneumophila* lysed by freeze-thaw lysed (FT-WT) probed with an ICDH-specific antiserum that recognizes a cytosolic *L*. *pneumophila* protein. Data represent the mean and standard deviation. *, *P* < 0.001; verses WT by two-tailed Student’s test.
(TIF)

S1 Table Tertiary structure predictions of ChiA N-terminal subdomains. (PDF)

S2 Table SAXS structural parameters. (PDF)

S3 Table SAXS ensemble optimization parameters. (PDF)

S4 Table Primers used in this study. (PDF)

S5 Table Synthetic genes. (PDF)
