# Supplementary figures and images for: Structure and functional analysis of the Legionella pneumophila chitinase ChiA reveals a novel mechanism of metal-dependent mucin degradation
Source: PLoS Pathog. 2020 May 4;16(5):e1008342. doi: 10.1371/journal.ppat.1008342 (PMC7224574; doi:10.1371/journal.ppat.1008342)

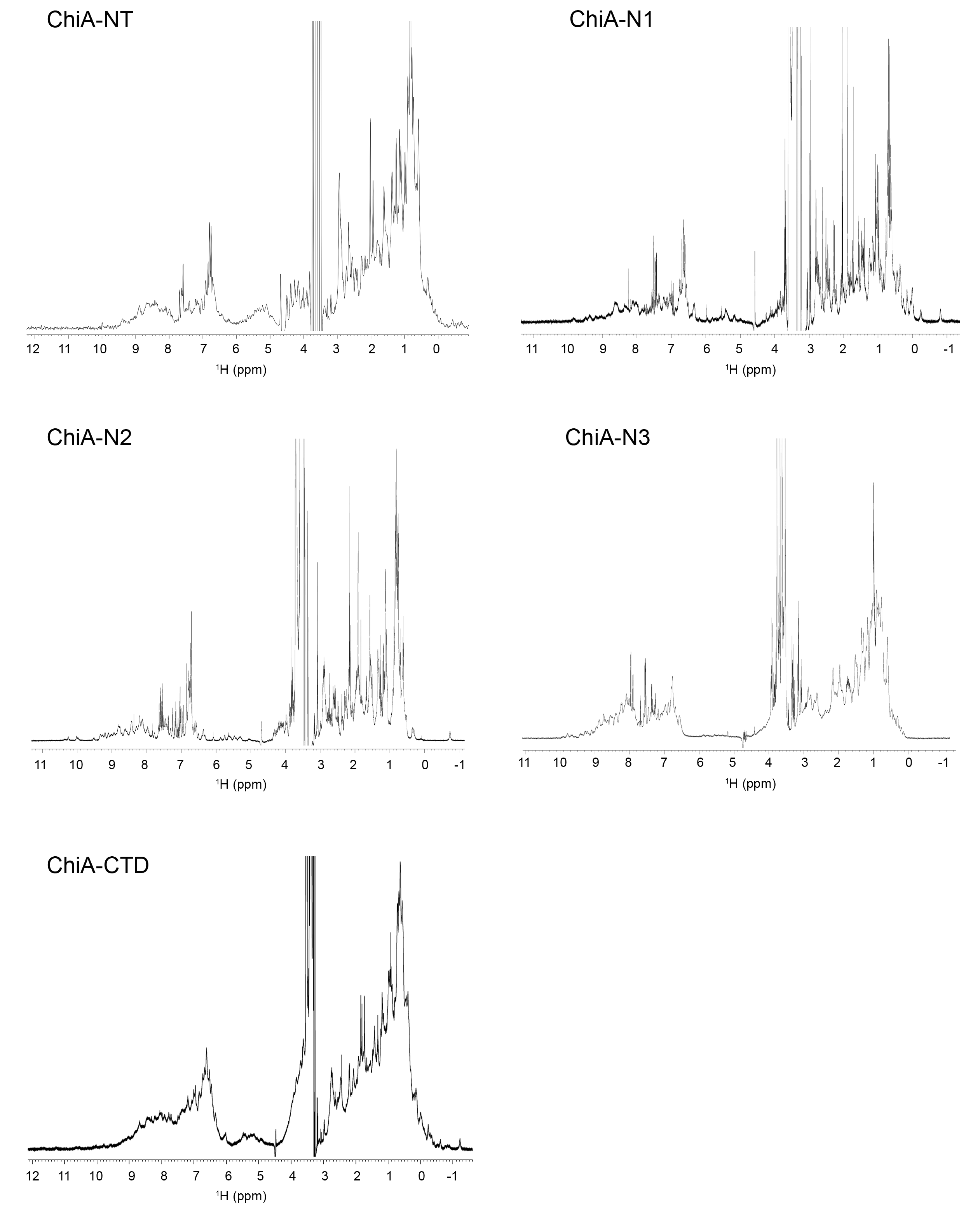

Supplement: S1 Fig — The methyl region of the NMR spectra includes high-field proton resonances observed at low chemical shifts (<0.5 ppm), which indicate the presence of characteristic clusters of aromatic and methyl groups in the core of a structured protein. In addition, the envelope of peaks resonating at high chemical shift (>8.5 ppm) correspond to highly ordered backbone amides present in secondary structure elements. (TIF) [file ppat.1008342.s001.tif]

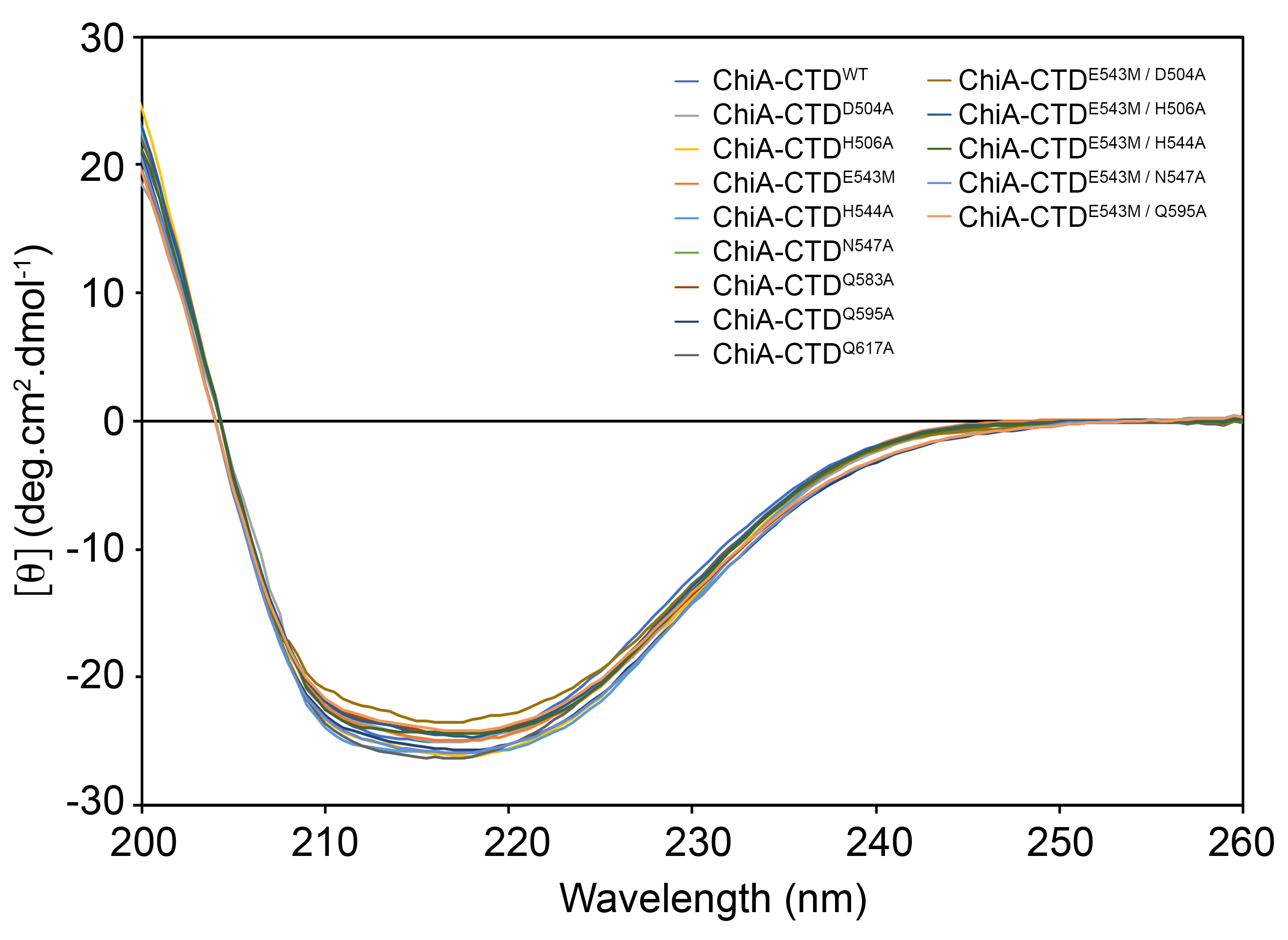

Supplement: S2 Fig — The negative bands between ~210 to ~ 220 nm and positive band at 200 nm is indicative of a mixed α/β protein fold. The spectra for wild-type (WT) ChiA-CTD and mutants are in essence identical and demonstrates that these mutations do not perturb the structure of the CTD domain. (TIF) [file ppat.1008342.s002.tif]

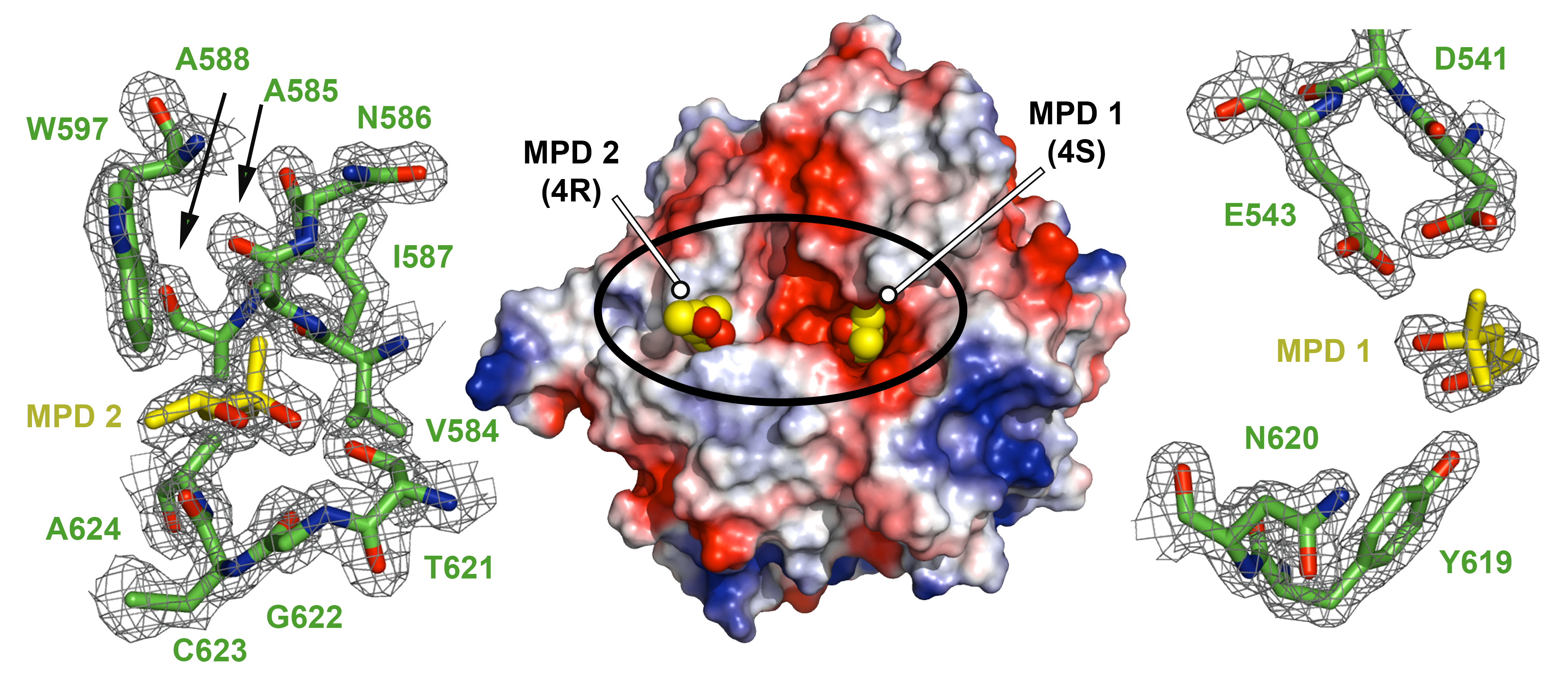

Supplement: S3 Fig — Electrostatic surface potential representation of ChiA-CTD with two molecules of MPD shown as spheres (MPD1: 4S enantiomer; MPD2: 4R enantiomer). Each binding site is expanded and the ρA weighted electron density maps contoured at 1.0 r.m.s. are shown. (TIF) [file ppat.1008342.s003.tif]

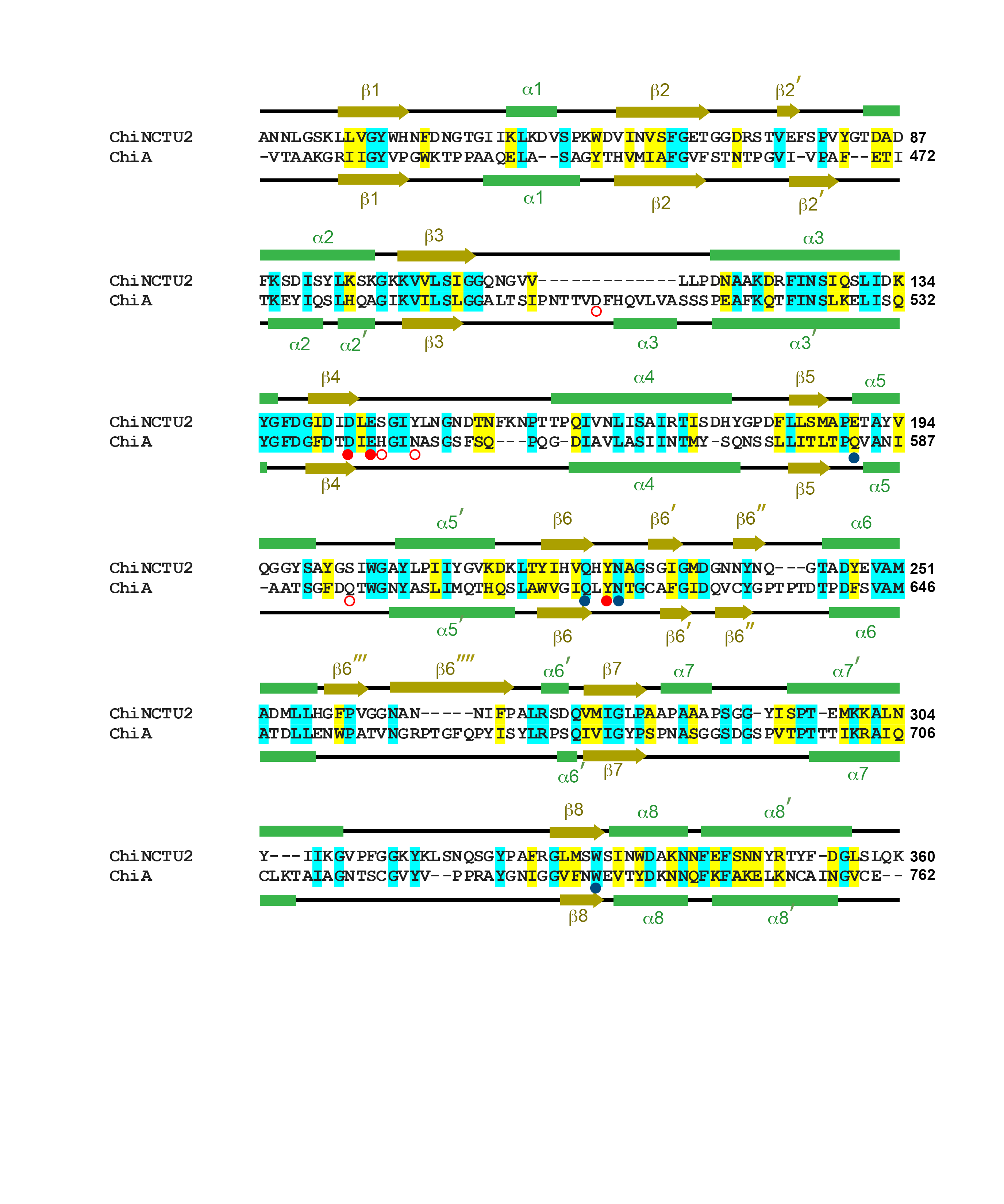

Supplement: S4 Fig — Secondary structure elements of ChiNCTU2 and ChiA-CTD are shown above and below, respectively (green rectangle: α-helix; gold arrow: β-strand). Amino acid identities and similar residues are indicated by background shading in cyan and yellow, respectively. Catalytic chitinase residues and chitin binding residues in ChiNCTU2 are indicated with red and blue filled circles, respectively. Mucinase active site residues in ChiA-CTD are shown as open red circles. (TIF) [file ppat.1008342.s004.tif]

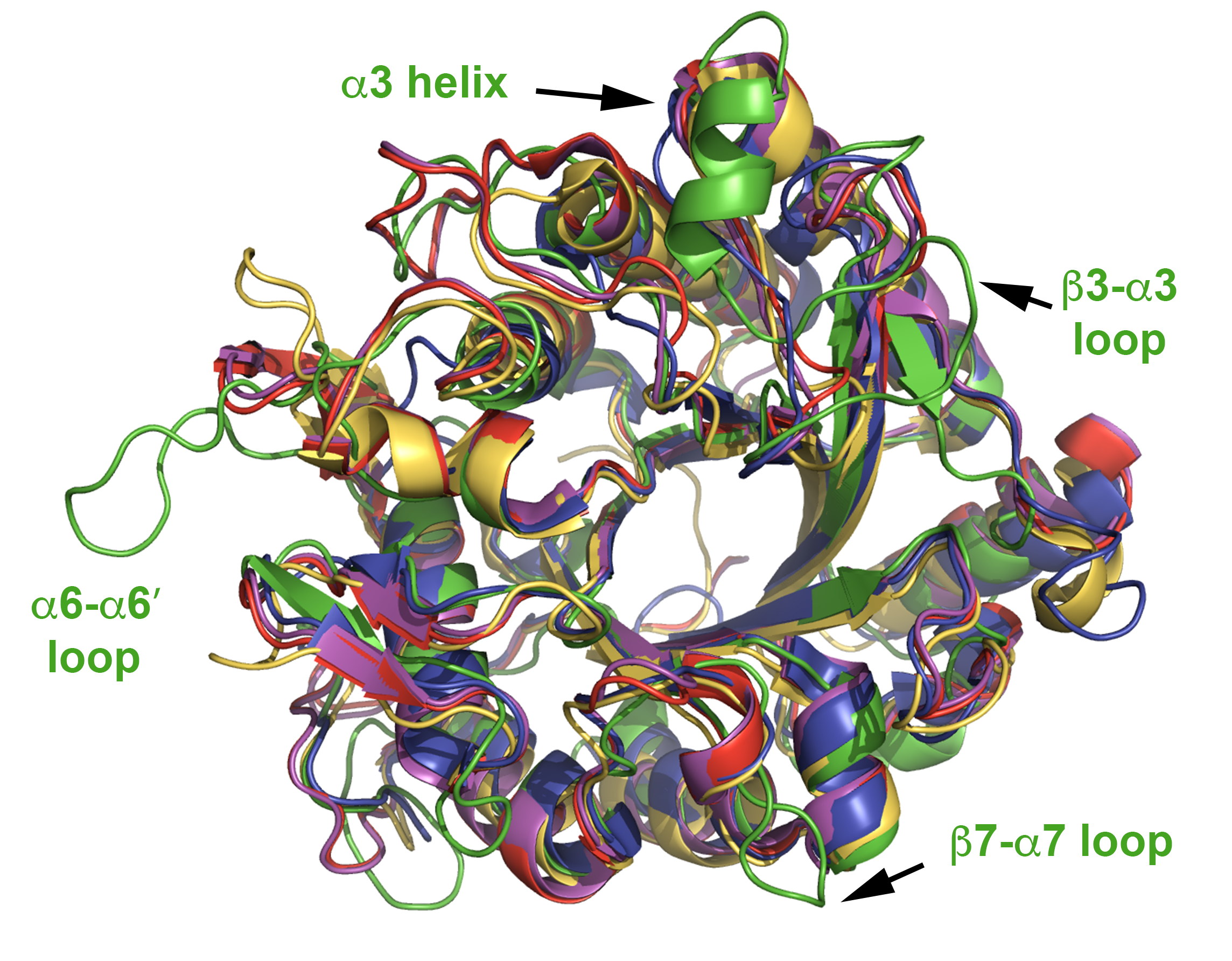

Supplement: S5 Fig — L. pneumophila ChiA-CTD is green, Bacillus cereus ChiNCTU2 is purple (PDB ID code 3n18) [27], Bacillus anthracis Chi36 is red (PDB ID code 5kz6, Chromobacterium violaceum ChiA is yellow (PDB ID code 4tx8) and Streptomyces coelicolor ChiA is blue (PDB ID code 3ebv). Augmented loop and helical structures in L. pneumophila ChiA-CTD are annotated. (TIF) [file ppat.1008342.s005.tif]

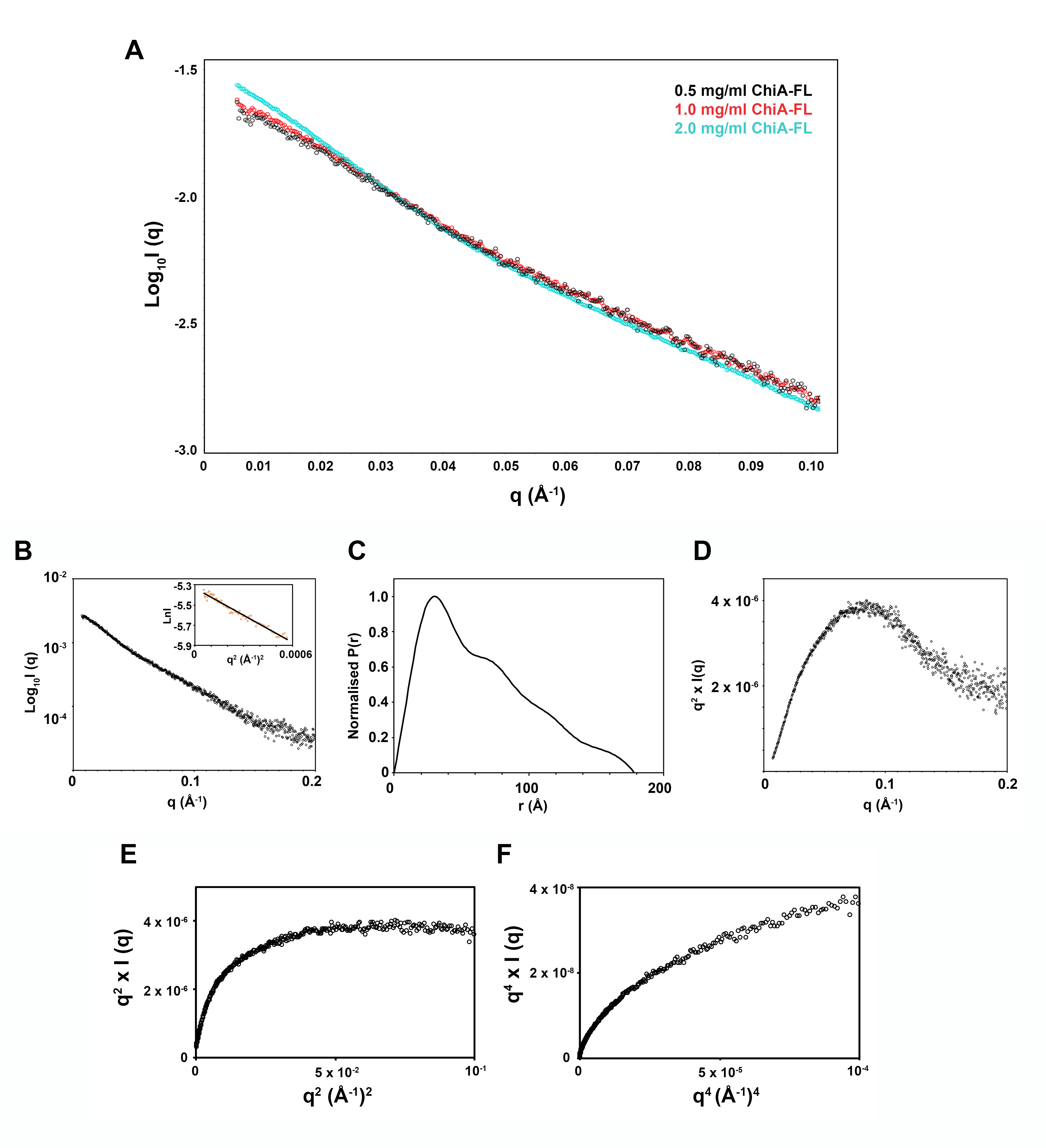

Supplement: S6 Fig — (A) Comparison of scaled scattering curves of ChiA-FL at 0.5 mg/ml (black), 1.0 mg/ml (red) and 2.0 mg/ml (teal) to highlight aggregation at concentrations above 1.0 mg/ml. (B) Experimental scattering curve of ChiA-FL (black open circles). Inset: Guinier Region (orange open circles) and linear regression (black line) for Rg evaluation. (C) Shape distribution [P(r)] function derived from SAXS analysis for ChiA. (D) Kratky, (E) Kratky-Debye and (F) Porod-Debye plots indicate that ChiA is a highly dynamic particle in solution. (TIF) [file ppat.1008342.s006.tif]

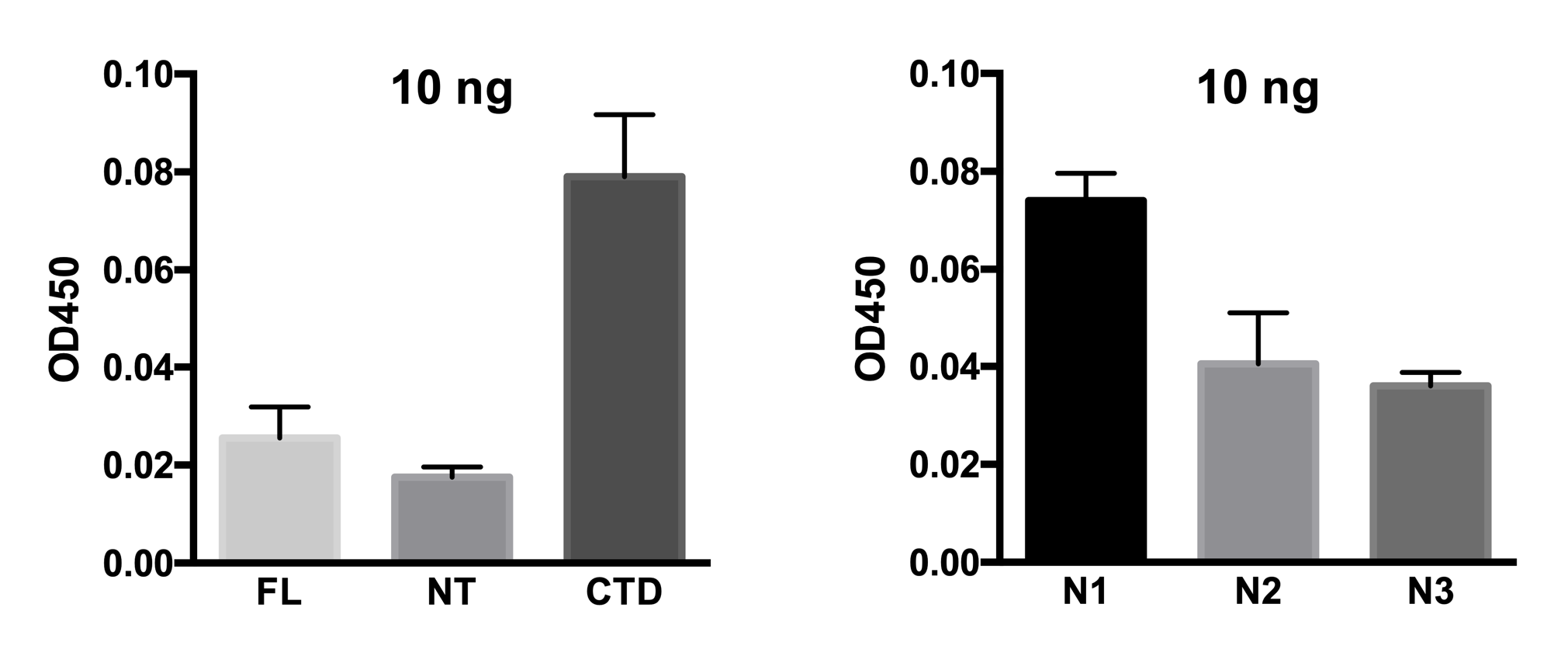

Supplement: S7 Fig — ELISA analysis of anti-ChiA antibodies binding to either full-length ChiA (FL), the N-terminal domain of ChiA (NT), and the C-terminal domain of ChiA (CTD) (left panel) or the ChiA N-terminal subdomain 1 (N1), subdomain 2 (N2), and subdomain 3 (N3) (right panel). All values represent the mean and standard deviation from triplicate wells. (TIF) [file ppat.1008342.s007.tif]

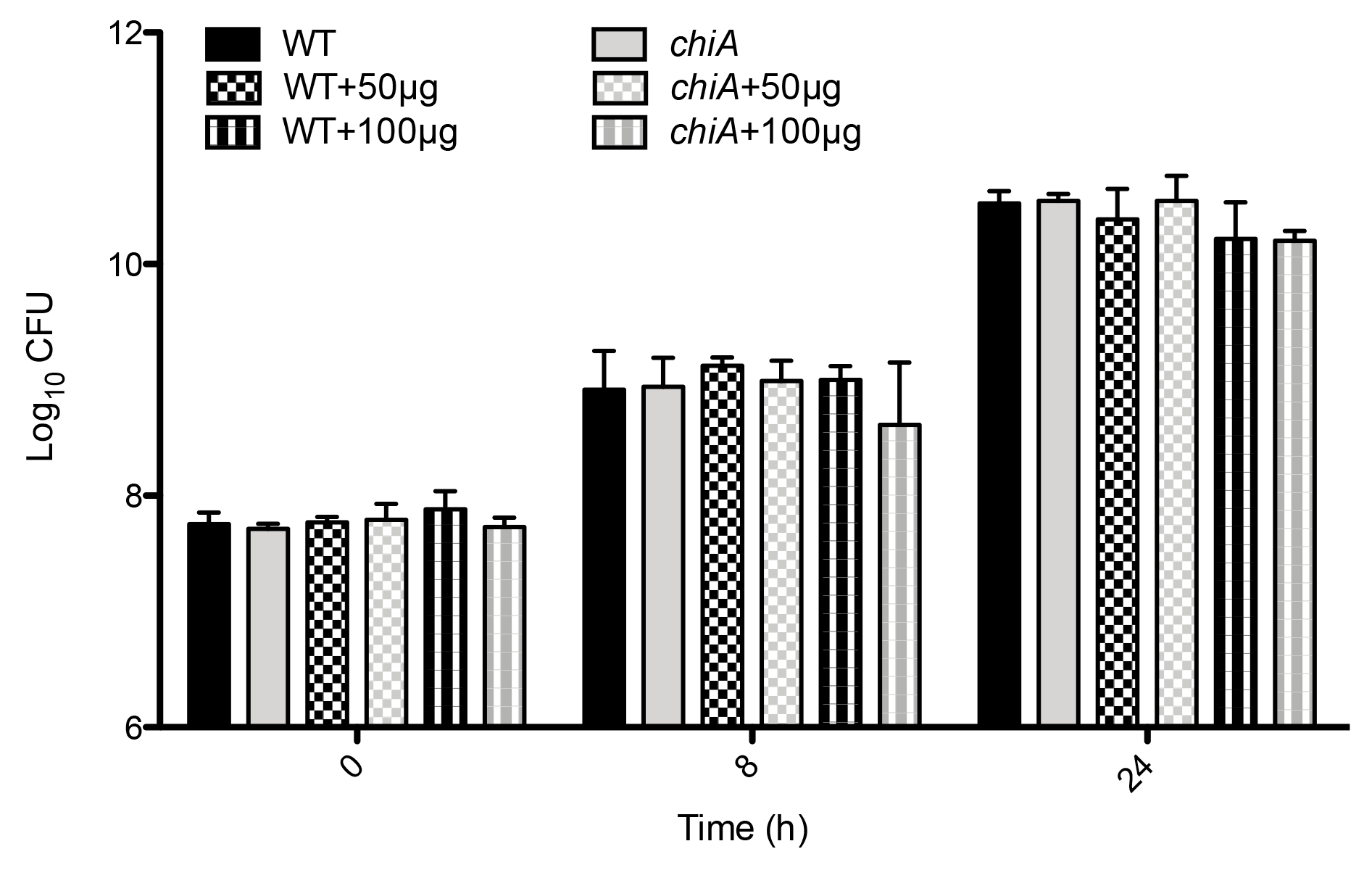

Supplement: S8 Fig — WT and chiA mutant bacteria were grown from a starting OD660 of 0.3 in chemically defined medium in the presence of porcine mucin II at the indicated concentrations. At 0 h, 8 h and 24 h, bacterial numbers were determined by plating for CFU. N = 3. Representative graph shown above as mean and standard deviation of technical replicates in triplicate. Two other experiments showed the same trends, with no significant difference between mutant or mucin effect. (TIF) [file ppat.1008342.s008.tif]

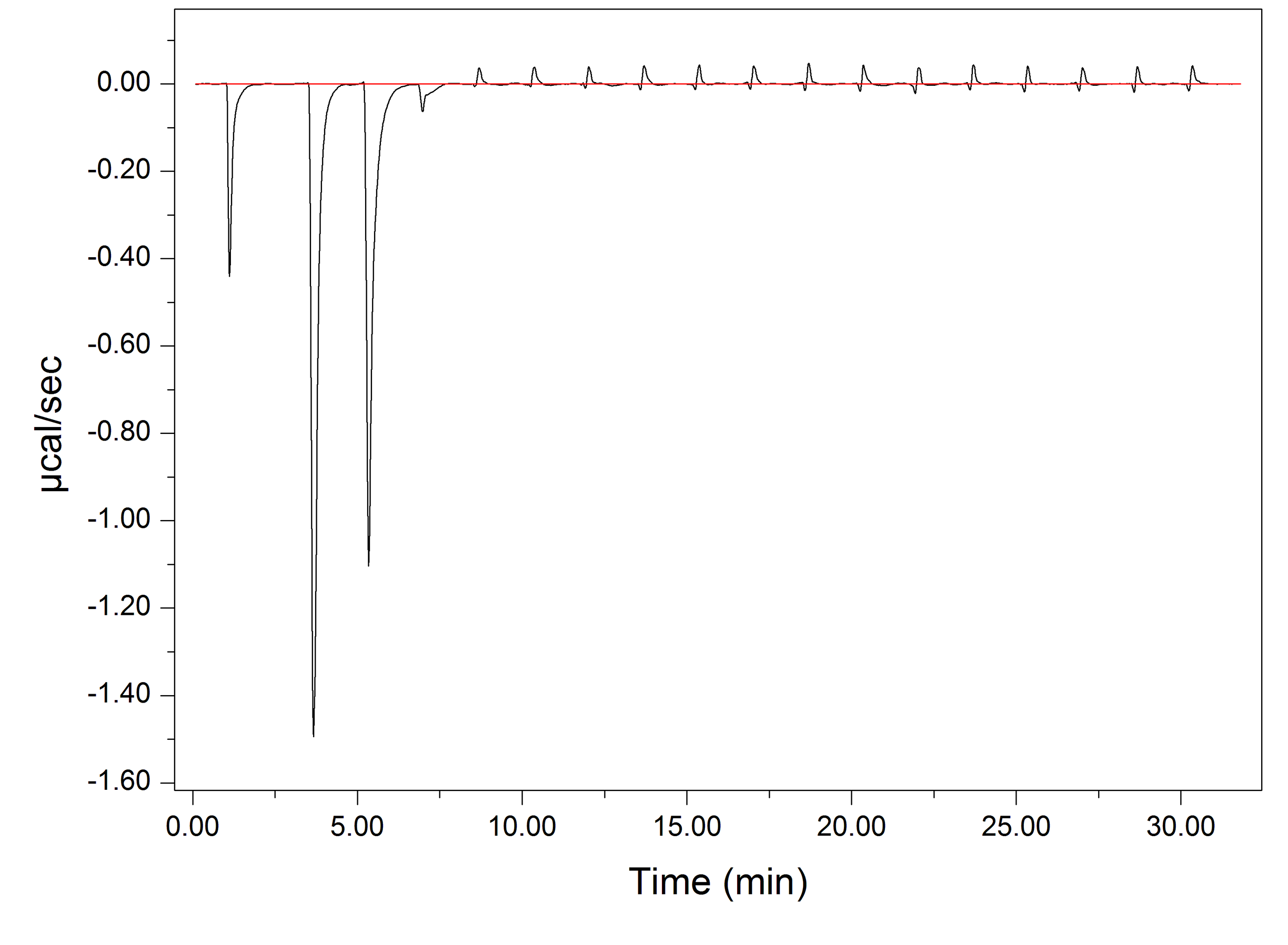

Supplement: S9 Fig — Titration of ChiA-CTD (syringe) into Zn2+ (cell) to assess heat generation during the dilution of ChiA-CTD. No significant heat generation was observed. (TIF) [file ppat.1008342.s009.tif]

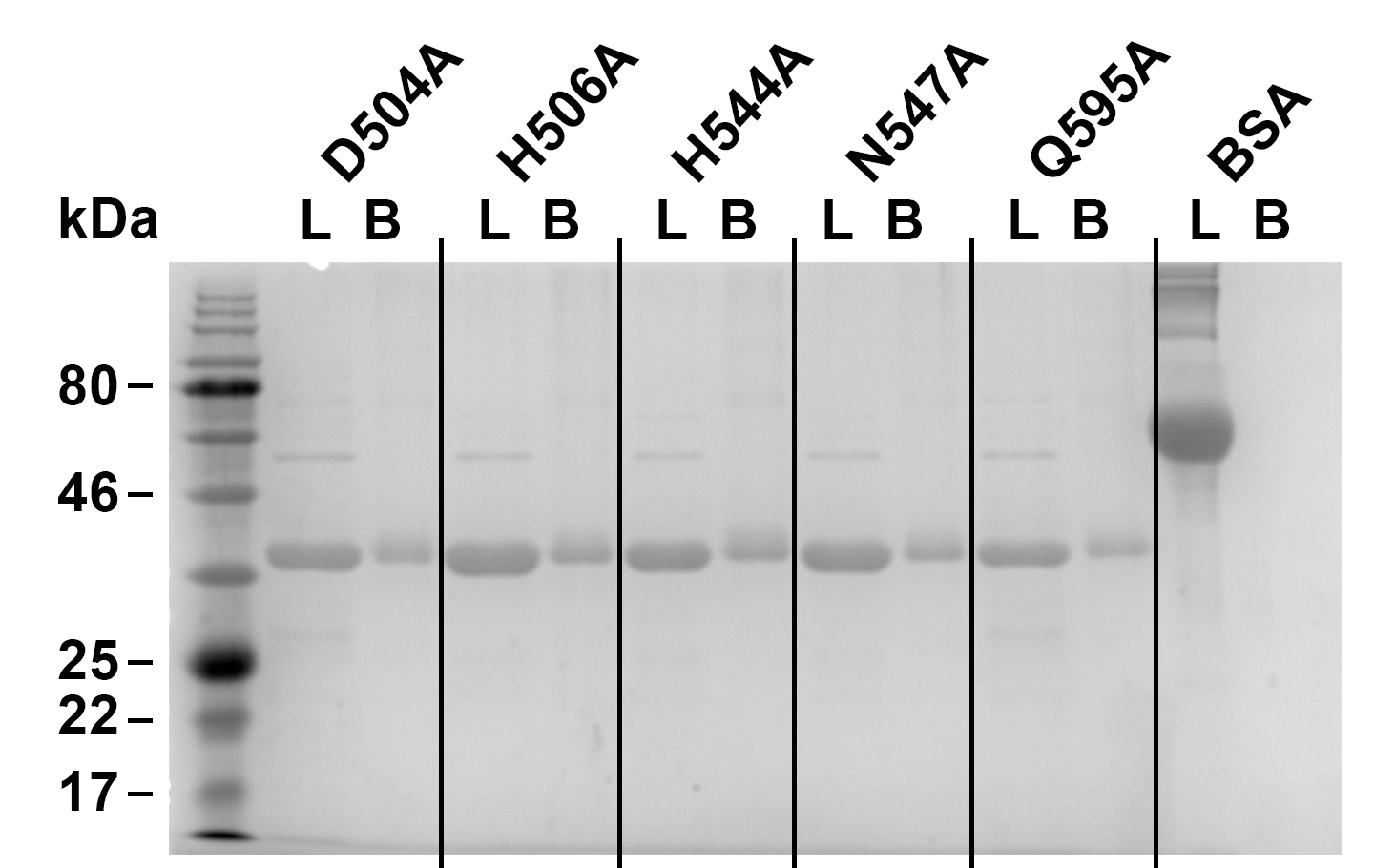

Supplement: S10 Fig — SDS-PAGE gels loaded with ChiA-CTD mutants or BSA control either before incubation with chitin beads (L) or after elution from the beads (B). Eluted samples undergo an upward shift compared to the input sample due to differences in buffer conditions. Data is representative of three independent repeat experiments. (TIF) [file ppat.1008342.s010.tif]

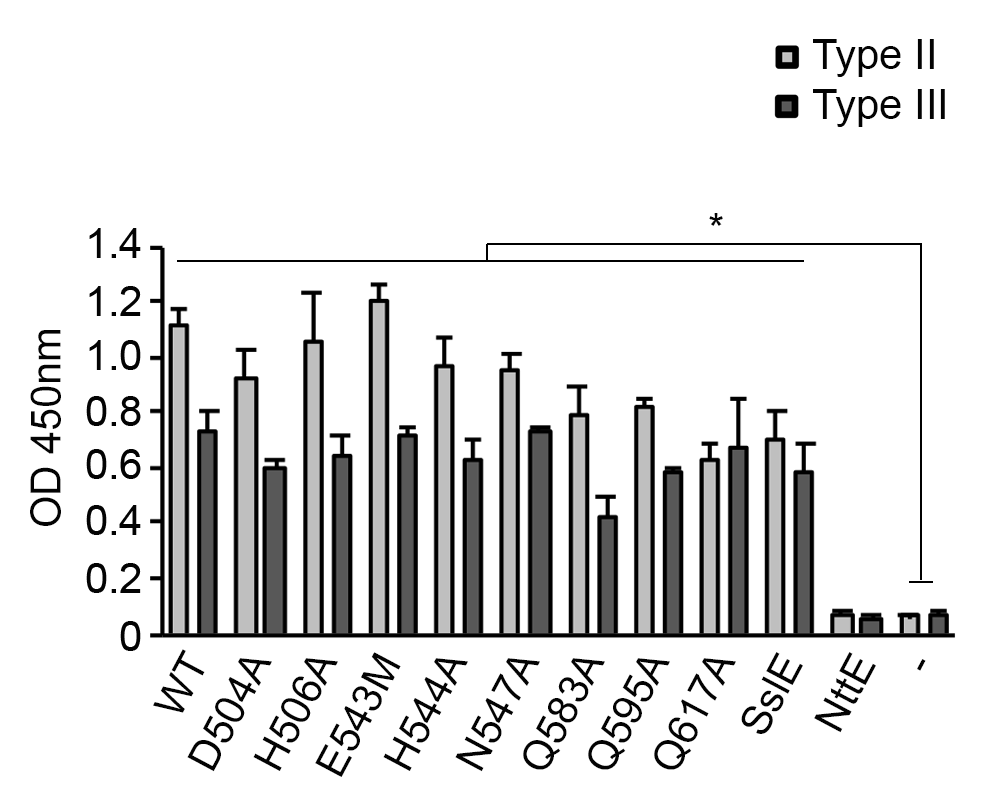

Supplement: S11 Fig — ELISA analysis of binding between immobilised type II or III mucin extracts and His-tagged wild-type ChiA-CTD (WT), ChiA-CTD mutants (D504A, H506A, E543M, H544A, N547A, Q583A, Q595A, 617A) and controls (SslE, NttE). Anti-His-tag antibody conjugated to HRP was used to measure OD450 nm values. BSA-coated wells were used as controls. Data represent the mean and standard deviation for triplicate experiments. *, P < 0.001; verses control empty well by two-tailed Student’s test. (TIF) [file ppat.1008342.s011.tif]

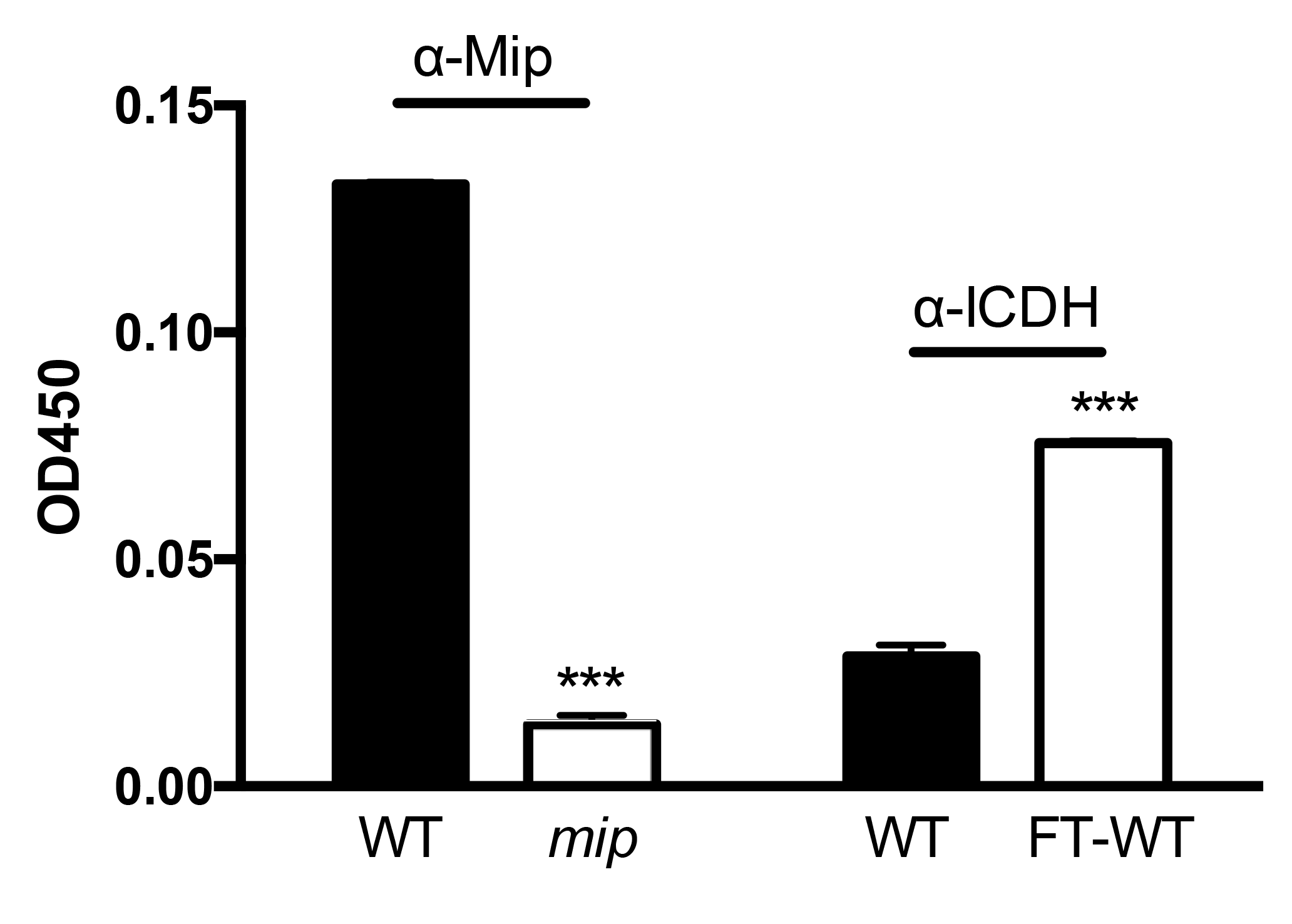

Supplement: S12 Fig — Whole cell ELISA of L. pneumophila wild-type 130b (WT) and mip mutant NU203 (mip) detected with Mip-specific antiserum, and L. pneumophila wild-type 130b (WT) and L. pneumophila lysed by freeze-thaw lysed (FT-WT) probed with an ICDH-specific antiserum that recognizes a cytosolic L. pneumophila protein. Data represent the mean and standard deviation. *, P < 0.001; verses WT by two-tailed Student’s test. (TIF) [file ppat.1008342.s012.tif]
