# Supplementary material for: Structure and functional analysis of the Legionella pneumophila chitinase ChiA reveals a novel mechanism of metal-dependent mucin degradation
Source: PLoS Pathog. 2020 May 4;16(5):e1008342. doi: 10.1371/journal.ppat.1008342 (PMC7224574; doi:10.1371/journal.ppat.1008342)
Supplement: S1 Table — (PDF) [file ppat.1008342.s013.pdf]

S1 Table. Tertiary structure predictions of ChiA N-terminal subdomains.

ChiA domain	Protein Name	Organism	Function	Sequence identity	PDB code	Ref	Prediction server
N1	RsgI2	*Clostridium thermocellum*	Putative cellulosomal family 3 carbohydrate binding module	16%	4B9P	[1]	Phyre2/Robetta
N1	ScaA	*Acetivibrio Cellulolyticus*	Family 3B carbohydrate binding module – binds strongly to cellulose	21%	3ZQW	[2]	Phyre2
N1	CipC	*Clostridium cellulolyticum*	Family 3A carbohydrate binding module – binds strongly to cellulose	20%	1G43	[3]	Phyre2
N1	Endo-glucanase D	*Clostridium cellulovorans*	C-terminal carbohydrate binding module	17%	3NDY	N/A	Phyre2
N1	Chi18aC	*Streptomyces coelicolor*	Chitinase chitin binding domain	18%	2RTT	N/A	Phyre2
N1	RsgI3	*Clostridium thermocellum*	Putative cellulosomal family 3 carbohydrate binding module	14%	4B97	[1]	Phyre2
N1	CBDcex	*Cellulomonas fimi*	Cellulose carbohydrate binding module	19%	1EXH	[4]	Phyre2
N2	RG-lyase	*Aspergillus aculeatus*	Rhamnogalacturonan lyase fibronectin type III domain	19%	1NKG	[5]	Phyre2
N2	CbhA	*Clostridium thermocellum*	Cellobiohydrolase A fibronectin type III domain	16%	3PE9/ 3PDG	[6]	Phyre2
N2	Tenascin	*Chicken*	Tandem fibronectin type III domains	12%	1QR4	[7]	Robetta
N3	ChiA	*Autographa californica nuclear polyhedrosis virus*	N-terminal chitinase domain – chitin binding	15%	5DF0	N/A	Phyre2

References

1. Yaniv O, Fichman G, Borovok I, Shoham Y, Bayer EA, Lamed R, et al. Fine-structural variance of family 3 carbohydrate-binding modules as extracellular biomass-sensing components of Clostridium thermocellum anti-sigmaI factors. Acta Crystallogr D Biol Crystallogr. 2014;70(Pt 2):522-34. Epub 2014/02/18. doi: 10.1107/S139900471302926X. PubMed PMID: 24531486.

2. Yaniv O, Halfon Y, Shimon LJ, Bayer EA, Lamed R, Frolow F. Structure of CBM3b of the major cellulosomal scaffoldin subunit ScaA from Acetivibrio cellulolyticus. Acta Crystallogr Sect F Struct Biol Cryst Commun. 2012;68(Pt 1):8-13. Epub 2012/01/11. doi: 10.1107/S174430911104807X. PubMed PMID: 22232162; PubMed Central PMCID: PMCPMC3253825.

3. Shimon LJ, Pages S, Belaich A, Belaich JP, Bayer EA, Lamed R, et al. Structure of a family IIIa scaffoldin CBD from the cellulosome of Clostridium cellulolyticum at 2.2 A resolution. Acta Crystallogr D Biol Crystallogr. 2000;56(Pt 12):1560-8. Epub 2000/11/28. PubMed PMID: 11092922.

4. Xu GY, Ong E, Gilkes NR, Kilburn DG, Muhandiram DR, Harris-Brandts M, et al. Solution structure of a cellulose-binding domain from Cellulomonas fimi by nuclear magnetic resonance spectroscopy. Biochemistry. 1995;34(21):6993-7009. Epub 1995/05/30. PubMed PMID: 7766609.

5. McDonough MA, Kadirvelraj R, Harris P, Poulsen JC, Larsen S. Rhamnogalacturonan lyase reveals a unique three-domain modular structure for polysaccharide lyase family 4. FEBS Lett. 2004;565(1-3):188-94. Epub 2004/05/12. doi: 10.1016/j.febslet.2004.03.094. PubMed PMID: 15135077.

6. Brunecky R, Alahuhta M, Bomble YJ, Xu Q, Baker JO, Ding SY, et al. Structure and function of the Clostridium thermocellum cellobiohydrolase A X1-module repeat: enhancement through stabilization of the CbhA complex. Acta Crystallogr D Biol Crystallogr. 2012;68(Pt 3):292-9. Epub 2012/02/22. doi: 10.1107/S0907444912001680. PubMed PMID: 22349231.

7. Bisig D, Weber P, Vaughan L, Winterhalter KH, Piontek K. Purification, crystallization and preliminary crystallographic studies of a two fibronectin type-III domain segment from chicken tenascin encompassing the heparin- and contactin-binding regions. Acta Crystallogr D Biol Crystallogr. 1999;55(Pt 5):1069-73. Epub 1999/04/27. PubMed PMID: 10216309.
