# Supplementary material for: Structure and functional analysis of the Legionella pneumophila chitinase ChiA reveals a novel mechanism of metal-dependent mucin degradation
Source: PLoS Pathog. 2020 May 4;16(5):e1008342. doi: 10.1371/journal.ppat.1008342 (PMC7224574; doi:10.1371/journal.ppat.1008342)
Supplement: S2 Table — (PDF) [file ppat.1008342.s014.pdf]

S2 Table. SAXS structural parameters.

SAXS data collection		
	Beamline	DLS B21
	Wavelength (Å)	1.0
	q Range ($Å^{-1}$)	0.004 to 0.4
	Concentration range (mg/mL)	0.5 to 4
Structural parameters		
	I(0)	5.36e-03 ± 2.6e-05
	R_g (nm) (from Guinier)	5.43 ± 0.05
	R_g (nm) (from P(r))	5.45 ± 0.03
	D_{max} (nm) (from P(r))	17.77
	MW (kDa) (from sequence)	82.6
	MW (kDa) (from SAXS)	89.2
