# Supplementary material for: Structure and functional analysis of the Legionella pneumophila chitinase ChiA reveals a novel mechanism of metal-dependent mucin degradation
Source: PLoS Pathog. 2020 May 4;16(5):e1008342. doi: 10.1371/journal.ppat.1008342 (PMC7224574; doi:10.1371/journal.ppat.1008342)
Supplement: S3 Table — (PDF) [file ppat.1008342.s015.pdf]

S3 Table. SAXS ensemble optimization parameters.

	Run 1	Run 2	Run 3	Average
R_{flex} (random)	90.47 (89.14)	91.60 (89.70)	92.14 (89.72)	91.4 (89.5)
R_{sig}	1.22	1.22	1.25	1.23
χ^2	1.07	1.05	1.14	1.09
Cluster 1 (%)	17.2	19.3	18.2	18.2
Cluster 2 (%)	49.4	40.6	46.4	45.5
Cluster 3 (%)	33.4	40.1	35.4	36.3
Final ensemble R_g (nm)	5.40	5.43	5.39	5.41
Final ensemble D_{max} (nm)	17.11	17.28	16.73	17.04
