# Supplementary material for: Structure and functional analysis of the Legionella pneumophila chitinase ChiA reveals a novel mechanism of metal-dependent mucin degradation
Source: PLoS Pathog. 2020 May 4;16(5):e1008342. doi: 10.1371/journal.ppat.1008342 (PMC7224574; doi:10.1371/journal.ppat.1008342)
Supplement: S4 Table — (PDF) [file ppat.1008342.s016.pdf]

S4 Table. Primers used in this study.

Primer	Description	Sequence (5' to 3')
JG9	ChiA-FL F	GACGACGACAAGATGAATACCTCTTTAAAACCCTCAATTGCC
JG10	ChiA-FL R	GAGGAGAAGCCCGGTTACTCACAAACACCATTAATAGCACAATTTTTC
JG9	ChiA-NT F	GACGACGACAAGATGAATACCTCTTTAAAACCCTCAATTGCC
JG11	ChiA-NT R	GAGGAGAAGCCCGGTTAAGATAAAGTGATGTTTACTGTTGCATTTTC
JG9	ChiA-N1 F	GACGACGACAAGATGAATACCTCTTTAAAACCCTCAATTGCC
JG12	ChiA-N1 R	GAGGAGAAGCCCGGTTAGATGCTTCCTGATTCAACCGTTG
JG13	ChiA-N2 F	GACGACGACAAGATGGGAAGCATCCAATTAATCAATGCTGC
JG14	ChiA-N2 R	GAGGAGAAGCCCGGTTAATCGTTATATTGAATTGCAGGAGTTGAG
JG15	ChiA-N3 F	GACGACGACAAGATGGGGAGTAGTTACCAATTCTCAACTC
JG11	ChiA-N3 R	GAGGAGAAGCCCGGTTAAGATAAAGTGATGTTTACTGTTGCATTTTC
JG16	ChiA-CTD F	GACGACGACAAGATGGTAACCGCTGCAAAAGGGC
JG10	ChiA-CTD R	GAGGAGAAGCCCGGTTACTCACAAACACCATTAATAGCACAATTTTTC
JG17	ChiA-CTD E543M mutant F	AGGAGTTAATTTCTCAATATGGTTTTGATGGGTTTGATACAGATATTATGCAT GGTATTAACGCTAGCGG
JG18	ChiA-CTD E543M mutant R	CCGCTAGCGTTAATACCATGCATAATATCTGTATCAAACCCATCAAAACCATA TTGAGAAATTAACTCCT
JG19	NttE F	GACGACGACAAGATGAATTCGGATGACAATGCTGATGGC
JG20	NttE R	GAGGAGAAGCCCGGTTACAAACAATGTTTATTAGGATTTCTCTTCATAC
PC1	SslE F	AGGAGATATACCATGAGCTACCCGCTCAACTATATG
PC2	SslE R	GTGATGGTGATGTTTCTCGACAGACATCTTATGC
