# Supplementary material for: Structure and functional analysis of the Legionella pneumophila chitinase ChiA reveals a novel mechanism of metal-dependent mucin degradation
Source: PLoS Pathog. 2020 May 4;16(5):e1008342. doi: 10.1371/journal.ppat.1008342 (PMC7224574; doi:10.1371/journal.ppat.1008342)
Supplement: S5 Table — (PDF) [file ppat.1008342.s017.pdf]

S5 Table. Synthetic genes.

Gene	Description	Sequence (5' to 3')
CM4	ChiA-CTD D504A mutant	CCATGGTCCATCATCATCATCATCATGTGGACGACGACGACAAGATGGTTACCGCAGCAAAAGG<br>TCGCATTATTGGTTATGTTCCAGGTTGGAAAACCCCTCCGGCAGCACAAGAACTGGCAAGCGCA<br>GGTTATACCCATGTTATGATTGCATTTGGTGTGTTCAGCACCAATACACCGGGTGTTATTGTTC<br>CGGCATTTGAAACCATTACCAAAGAGTATATTCAGAGCCTGCATCAGGCAGGCATTAAAGTTAT<br>TCTGAGCTTAGGTGGTGCACTGACCAGCATTCCGAATACCACCGTTGCGTTTCATCAGGTTCTG<br>GTTGCAAGCAGCAGTCCGGAAGCATTTAAACAGACCTTTATTAACAGCCTGAAAGAACTGATTA<br>GCCAGTATGGTTTTGATGGCTTTGATACCGATATTGAGCATGGTATTAATGCCAGCGGTAGCTT<br>TAGCCAGCCGCAGGGTGATATTGCAGTTCTGGCAAGCATTATTAACACCATGTATAGCCAGAAT<br>AGCAGCCTGCTGATTACCCTGACACCGCAGGTTGCAAATATTGCAGCAACCAGCGGTTTTGATC<br>AGACCTGGGGTAATTATGCAAGCCTGATTATGCAGACCCATCAGAGTCTGGCATGGGTTGGTAT<br>TCAGCTGTATAATACCGGTTGTGCCTTTGGTATTGATCAGGTTTGTTATGGTCCGACACCGACC<br>GATACACCGGATTTTAGCGTTGCAATGGCAACCGATCTGCTGGAAAATTGGCCTGCAACCGTTA<br>ATGGTCGTCCGACCGGTTTTCAGCCGTATATTAGCTATCTGCGTCCGAGCCAGATTGTGATTGG<br>TTATCCGAGTCCGAATGCAAGCGGTGGTAGTGATGGTAGTCCGGTTACACCGACCACCACCATT<br>AAACGTGCAATTCAGTGTCTGAAAACAGCCATTGCAGGTAATACCAGCTGTGGTGTTTATGTTC<br>CGCCTCGTGCATATGGTAATATTGGTGGTGTGTTTAATTGGGAAGTGACCTACGATAAAAACAA<br>CCAGTTCAAATTCGCCAAAGAGCTGAAAAATTGCGCGATTAATGGCGTGTGTGAATAACTCGAG
CM5	ChiA-CTD H506A mutant	CCATGGTCCATCATCATCATCATCATGTGGACGACGACGACAAGATGGTTACCGCAGCAAAAGG<br>TCGCATTATTGGTTATGTTCCAGGTTGGAAAACCCCTCCGGCAGCACAAGAACTGGCAAGCGCA<br>GGTTATACCCATGTTATGATTGCATTTGGTGTGTTCAGCACCAATACACCGGGTGTTATTGTTC<br>CGGCATTTGAAACCATTACCAAAGAGTATATTCAGAGCCTGCATCAGGCAGGCATTAAAGTTAT<br>TCTGAGCTTAGGTGGTGCACTGACCAGCATTCCGAATACCACCGTTGATTTTGCGCAGGTTCTG<br>GTTGCAAGCAGCAGTCCGGAAGCATTTAAACAGACCTTTATTAACAGCCTGAAAGAACTGATTA<br>GCCAGTATGGTTTTGATGGCTTTGATACCGATATTGAGCATGGTATTAATGCCAGCGGTAGCTT<br>TAGCCAGCCGCAGGGTGATATTGCAGTTCTGGCAAGCATTATTAACACCATGTATAGCCAGAAT<br>AGCAGCCTGCTGATTACCCTGACACCGCAGGTTGCAAATATTGCAGCAACCAGCGGTTTTGATC<br>AGACCTGGGGTAATTATGCAAGCCTGATTATGCAGACCCATCAGAGTCTGGCATGGGTTGGTAT<br>TCAGCTGTATAATACCGGTTGTGCCTTTGGTATTGATCAGGTTTGTTATGGTCCGACACCGACC<br>GATACACCGGATTTTAGCGTTGCAATGGCAACCGATCTGCTGGAAAATTGGCCTGCAACCGTTA<br>ATGGTCGTCCGACCGGTTTTCAGCCGTATATTAGCTATCTGCGTCCGAGCCAGATTGTGATTGG<br>TTATCCGAGTCCGAATGCAAGCGGTGGTAGTGATGGTAGTCCGGTTACACCGACCACCACCATT<br>AAACGTGCAATTCAGTGTCTGAAAACAGCCATTGCAGGTAATACCAGCTGTGGTGTTTATGTTC<br>CGCCTCGTGCATATGGTAATATTGGTGGTGTGTTTAATTGGGAAGTGACCTACGATAAAAACAA<br>CCAGTTCAAATTCGCCAAAGAGCTGAAAAATTGCGCGATTAATGGCGTGTGTGAATAACTCGAG
CM6	ChiA-CTD H544A mutant	CCATGGTCCATCATCATCATCATCATGTGGACGACGACGACAAGATGGTTACCGCAGCAAAAGG<br>TCGCATTATTGGTTATGTTCCAGGTTGGAAAACCCCTCCGGCAGCACAAGAACTGGCAAGCGCA<br>GGTTATACCCATGTTATGATTGCATTTGGTGTGTTCAGCACCAATACACCGGGTGTTATTGTTC<br>CGGCATTTGAAACCATTACCAAAGAGTATATTCAGAGCCTGCATCAGGCAGGCATTAAAGTTAT<br>TCTGAGCTTAGGTGGTGCACTGACCAGCATTCCGAATACCACCGTTGATTTTCATCAGGTTCTG<br>GTTGCAAGCAGCAGTCCGGAAGCATTTAAACAGACCTTTATTAACAGCCTGAAAGAACTGATTA<br>GCCAGTATGGTTTTGATGGCTTTGATACCGATATTGAGGCGGGTATTAATGCCAGCGGTAGCTT<br>TAGCCAGCCGCAGGGTGATATTGCAGTTCTGGCAAGCATTATTAACACCATGTATAGCCAGAAT<br>AGCAGCCTGCTGATTACCCTGACACCGCAGGTTGCAAATATTGCAGCAACCAGCGGTTTTGATC<br>AGACCTGGGGTAATTATGCAAGCCTGATTATGCAGACCCATCAGAGTCTGGCATGGGTTGGTAT<br>TCAGCTGTATAATACCGGTTGTGCCTTTGGTATTGATCAGGTTTGTTATGGTCCGACACCGACC<br>GATACACCGGATTTTAGCGTTGCAATGGCAACCGATCTGCTGGAAAATTGGCCTGCAACCGTTA<br>ATGGTCGTCCGACCGGTTTTCAGCCGTATATTAGCTATCTGCGTCCGAGCCAGATTGTGATTGG<br>TTATCCGAGTCCGAATGCAAGCGGTGGTAGTGATGGTAGTCCGGTTACACCGACCACCACCATT<br>AAACGTGCAATTCAGTGTCTGAAAACAGCCATTGCAGGTAATACCAGCTGTGGTGTTTATGTTC<br>CGCCTCGTGCATATGGTAATATTGGTGGTGTGTTTAATTGGGAAGTGACCTACGATAAAAACAA<br>CCAGTTCAAATTCGCCAAAGAGCTGAAAAATTGCGCGATTAATGGCGTGTGTGAATAACTCGAG
CM7	ChiA-CTD N547A mutant	CCATGGTCCATCATCATCATCATCATGTGGACGACGACGACAAGATGGTTACCGCAGCAAAAGG<br>TCGCATTATTGGTTATGTTCCAGGTTGGAAAACCCCTCCGGCAGCACAAGAACTGGCAAGCGCA<br>GGTTATACCCATGTTATGATTGCATTTGGTGTGTTCAGCACCAATACACCGGGTGTTATTGTTC<br>CGGCATTTGAAACCATTACCAAAGAGTATATTCAGAGCCTGCATCAGGCAGGCATTAAAGTTAT<br>TCTGAGCTTAGGTGGTGCACTGACCAGCATTCCGAATACCACCGTTGATTTTCATCAGGTTCTG<br>GTTGCAAGCAGCAGTCCGGAAGCATTTAAACAGACCTTTATTAACAGCCTGAAAGAACTGATTA<br>GCCAGTATGGTTTTGATGGCTTTGATACCGATATTGAGCATGGTATTGCGGCCAGCGGTAGCTT<br>TAGCCAGCCGCAGGGTGATATTGCAGTTCTGGCAAGCATTATTAACACCATGTATAGCCAGAAT<br>AGCAGCCTGCTGATTACCCTGACACCGCAGGTTGCAAATATTGCAGCAACCAGCGGTTTTGATC<br>AGACCTGGGGTAATTATGCAAGCCTGATTATGCAGACCCATCAGAGTCTGGCATGGGTTGGTAT<br>TCAGCTGTATAATACCGGTTGTGCCTTTGGTATTGATCAGGTTTGTTATGGTCCGACACCGACC<br>GATACACCGGATTTTAGCGTTGCAATGGCAACCGATCTGCTGGAAAATTGGCCTGCAACCGTTA

		ATGGTCGTCCGACCGGTTTTCAGCCGTATATTAGCTATCTGCGTCCGAGCCAGATTGTGATTGG<br>TTATCCGAGTCCGAATGCAAGCGGTGGTAGTGATGGTAGTCCGGTTACACCGACCACCACCATT<br>AAACGTGCAATTCAGTGTCTGAAAACAGCCATTGCAGGTAATACCAGCTGTGGTGTTTATGTTC<br>CGCCTCGTGCATATGGTAATATTGGTGGTGTGTTTAATTGGGAAGTGACCTACGATAAAAACAA<br>CCAGTTCAAATTCGCCAAAGAGCTGAAAAATTGCGCGATTAATGGCGTGTGTGAATAACTCGAG
CM8	ChiA-CTD Q595A mutant	CCATGGTCCATCATCATCATCATCATGTGGACGACGACGACAAGATGGTTACCGCAGCAAAAGG<br>TCGCATTATTGGTTATGTTCCAGGTTGGAAAACCCCTCCGGCAGCACAAGAACTGGCAAGCGCA<br>GGTTATACCCATGTTATGATTGCATTTGGTGTGTTCAGCACCAATACACCGGGTGTTATTGTTC<br>CGGCATTTGAAACCATTACCAAAGAGTATATTCAGAGCCTGCATCAGGCAGGCATTAAAGTTAT<br>TCTGAGCTTAGGTGGTGCACTGACCAGCATTCCGAATACCACCGTTGATTTTCATCAGGTTCTG<br>GTTGCAAGCAGCAGTCCGGAAGCATTTAAACAGACCTTTATTAACAGCCTGAAAGAACTGATTA<br>GCCAGTATGGTTTTGATGGCTTTGATACCGATATTGAGCATGGTATTAATGCCAGCGGTAGCTT<br>TAGCCAGCCGCAGGGTGATATTGCAGTTCTGGCAAGCATTATTAACACCATGTATAGCCAGAAT<br>AGCAGCCTGCTGATTACCCTGACACCGCAGGTTGCAAATATTGCAGCAACCAGCGGTTTTGATG<br>CGACCTGGGGTAATTATGCAAGCCTGATTATGCAGACCCATCAGAGTCTGGCATGGGTTGGTAT<br>TCAGCTGTATAATACCGGTTGTGCCTTTGGTATTGATCAGGTTTGTTATGGTCCGACACCGACC<br>GATACACCGGATTTTAGCGTTGCAATGGCAACCGATCTGCTGGAAAATTGGCCTGCAACCGTTA<br>ATGGTCGTCCGACCGGTTTTCAGCCGTATATTAGCTATCTGCGTCCGAGCCAGATTGTGATTGG<br>TTATCCGAGTCCGAATGCAAGCGGTGGTAGTGATGGTAGTCCGGTTACACCGACCACCACCATT<br>AAACGTGCAATTCAGTGTCTGAAAACAGCCATTGCAGGTAATACCAGCTGTGGTGTTTATGTTC<br>CGCCTCGTGCATATGGTAATATTGGTGGTGTGTTTAATTGGGAAGTGACCTACGATAAAAACAA<br>CCAGTTCAAATTCGCCAAAGAGCTGAAAAATTGCGCGATTAATGGCGTGTGTGAATAACTCGAG
CM9	ChiA-CTD Q583A mutant	CCATGGTCCATCATCATCATCATCATGTGGACGACGACGACAAGATGGTTACCGCAGCAAAAGG<br>TCGCATTATTGGTTATGTTCCAGGTTGGAAAACCCCTCCGGCAGCACAAGAACTGGCAAGCGCA<br>GGTTATACCCATGTTATGATTGCATTTGGTGTGTTCAGCACCAATACACCGGGTGTTATTGTTC<br>CGGCATTTGAAACCATTACCAAAGAGTATATTCAGAGCCTGCATCAGGCAGGCATTAAAGTTAT<br>TCTGAGCTTAGGTGGTGCACTGACCAGCATTCCGAATACCACCGTTGATTTTCATCAGGTTCTG<br>GTTGCAAGCAGCAGTCCGGAAGCATTTAAACAGACCTTTATTAACAGCCTGAAAGAACTGATTA<br>GCCAGTATGGTTTTGATGGCTTTGATACCGATATTGAGCATGGTATTAATGCCAGCGGTAGCTT<br>TAGCCAGCCGCAGGGTGATATTGCAGTTCTGGCAAGCATTATTAACACCATGTATAGCCAGAAT<br>AGCAGCCTGCTGATTACCCTGACACCGGCGGTTGCAAATATTGCAGCAACCAGCGGTTTTGATC<br>AGACCTGGGGTAATTATGCAAGCCTGATTATGCAGACCCATCAGAGTCTGGCATGGGTTGGTAT<br>TCAGCTGTATAATACCGGTTGTGCCTTTGGTATTGATCAGGTTTGTTATGGTCCGACACCGACC<br>GATACACCGGATTTTAGCGTTGCAATGGCAACCGATCTGCTGGAAAATTGGCCTGCAACCGTTA<br>ATGGTCGTCCGACCGGTTTTCAGCCGTATATTAGCTATCTGCGTCCGAGCCAGATTGTGATTGG<br>TTATCCGAGTCCGAATGCAAGCGGTGGTAGTGATGGTAGTCCGGTTACACCGACCACCACCATT<br>AAACGTGCAATTCAGTGTCTGAAAACAGCCATTGCAGGTAATACCAGCTGTGGTGTTTATGTTC<br>CGCCTCGTGCATATGGTAATATTGGTGGTGTGTTTAATTGGGAAGTGACCTACGATAAAAACAA<br>CCAGTTCAAATTCGCCAAAGAGCTGAAAAATTGCGCGATTAATGGCGTGTGTGAATAACTCGAG
CM10	ChiA-CTD Q617A mutant	CCATGGTCCATCATCATCATCATCATGTGGACGACGACGACAAGATGGTTACCGCAGCAAAAGG<br>TCGCATTATTGGTTATGTTCCAGGTTGGAAAACCCCTCCGGCAGCACAAGAACTGGCAAGCGCA<br>GGTTATACCCATGTTATGATTGCATTTGGTGTGTTCAGCACCAATACACCGGGTGTTATTGTTC<br>CGGCATTTGAAACCATTACCAAAGAGTATATTCAGAGCCTGCATCAGGCAGGCATTAAAGTTAT<br>TCTGAGCTTAGGTGGTGCACTGACCAGCATTCCGAATACCACCGTTGATTTTCATCAGGTTCTG<br>GTTGCAAGCAGCAGTCCGGAAGCATTTAAACAGACCTTTATTAACAGCCTGAAAGAACTGATTA<br>GCCAGTATGGTTTTGATGGCTTTGATACCGATATTGAGCATGGTATTAATGCCAGCGGTAGCTT<br>TAGCCAGCCGCAGGGTGATATTGCAGTTCTGGCAAGCATTATTAACACCATGTATAGCCAGAAT<br>AGCAGCCTGCTGATTACCCTGACACCGCAGGTTGCAAATATTGCAGCAACCAGCGGTTTTGATC<br>AGACCTGGGGTAATTATGCAAGCCTGATTATGCAGACCCATCAGAGTCTGGCATGGGTTGGTAT<br>TGCGCTGTATAATACCGGTTGTGCCTTTGGTATTGATCAGGTTTGTTATGGTCCGACACCGACC<br>GATACACCGGATTTTAGCGTTGCAATGGCAACCGATCTGCTGGAAAATTGGCCTGCAACCGTTA<br>ATGGTCGTCCGACCGGTTTTCAGCCGTATATTAGCTATCTGCGTCCGAGCCAGATTGTGATTGG<br>TTATCCGAGTCCGAATGCAAGCGGTGGTAGTGATGGTAGTCCGGTTACACCGACCACCACCATT<br>AAACGTGCAATTCAGTGTCTGAAAACAGCCATTGCAGGTAATACCAGCTGTGGTGTTTATGTTC<br>CGCCTCGTGCATATGGTAATATTGGTGGTGTGTTTAATTGGGAAGTGACCTACGATAAAAACAA<br>CCAGTTCAAATTCGCCAAAGAGCTGAAAAATTGCGCGATTAATGGCGTGTGTGAATAACTCGAG
CM11	ChiA-CTD E543M/ D504A mutant	CCATGGTCCATCATCATCATCATCATGTGGACGACGACGACAAGATGGTTACCGCAGCAAAAGG<br>TCGCATTATTGGTTATGTTCCAGGTTGGAAAACCCCTCCGGCAGCACAAGAACTGGCAAGCGCA<br>GGTTATACCCATGTTATGATTGCATTTGGTGTGTTCAGCACCAATACACCGGGTGTTATTGTTC<br>CGGCATTTGAAACCATTACCAAAGAGTATATTCAGAGCCTGCATCAGGCAGGCATTAAAGTTAT<br>TCTGAGCTTAGGTGGTGCACTGACCAGCATTCCGAATACCACCGTTGCGTTTCATCAGGTTCTG<br>GTTGCAAGCAGCAGTCCGGAAGCATTTAAACAGACCTTTATTAACAGCCTGAAAGAACTGATTA<br>GCCAGTATGGTTTTGATGGCTTTGATACCGATATTATGCATGGTATTAATGCCAGCGGTAGCTT<br>TAGCCAGCCGCAGGGTGATATTGCAGTTCTGGCAAGCATTATTAACACCATGTATAGCCAGAAT<br>AGCAGCCTGCTGATTACCCTGACACCGCAGGTTGCAAATATTGCAGCAACCAGCGGTTTTGATC<br>AGACCTGGGGTAATTATGCAAGCCTGATTATGCAGACCCATCAGAGTCTGGCATGGGTTGGTAT<br>TCAGCTGTATAATACCGGTTGTGCCTTTGGTATTGATCAGGTTTGTTATGGTCCGACACCGACC

		GATACACCGGATTTTAGCGTTGCAATGGCAACCGATCTGCTGGAAAATTGGCCTGCAACCGTTA<br>ATGGTCGTCCGACCGGTTTTCAGCCGTATATTAGCTATCTGCGTCCGAGCCAGATTGTGATTGG<br>TTATCCGAGTCCGAATGCAAGCGGTGGTAGTGATGGTAGTCCGGTTACACCGACCACCACCATT<br>AAACGTGCAATTCAGTGTCTGAAAACAGCCATTGCAGGTAATACCAGCTGTGGTGTTTATGTTC<br>CGCCTCGTGCATATGGTAATATTGGTGGTGTGTTTAATTGGGAAGTGACCTACGATAAAAACAA<br>CCAGTTCAAATTCGCCAAAGAGCTGAAAAATTGCGCGATTAATGGCGTGTGTGAATAACTCGAG
CM12	ChiA-CTD E543M/ H506A mutant	CCATGGTCCATCATCATCATCATCATGTGGACGACGACGACAAGATGGTTACCGCAGCAAAAGG<br>TCGCATTATTGGTTATGTTCCAGGTTGGAAAACCCCTCCGGCAGCACAAGAACTGGCAAGCGCA<br>GGTTATACCCATGTTATGATTGCATTTGGTGTGTTCAGCACCAATACACCGGGTGTTATTGTTC<br>CGGCATTTGAAACCATTACCAAAGAGTATATTCAGAGCCTGCATCAGGCAGGCATTAAAGTTAT<br>TCTGAGCTTAGGTGGTGCACTGACCAGCATTCCGAATACCACCGTTGATTTTGCGCAGGTTCTG<br>GTTGCAAGCAGCAGTCCGGAAGCATTTAAACAGACCTTTATTAACAGCCTGAAAGAACTGATTA<br>GCCAGTATGGTTTTGATGGCTTTGATACCGATATTATGCATGGTATTAATGCCAGCGGTAGCTT<br>TAGCCAGCCGCAGGGTGATATTGCAGTTCTGGCAAGCATTATTAACACCATGTATAGCCAGAAT<br>AGCAGCCTGCTGATTACCCTGACACCGCAGGTTGCAAATATTGCAGCAACCAGCGGTTTTGATC<br>AGACCTGGGGTAATTATGCAAGCCTGATTATGCAGACCCATCAGAGTCTGGCATGGGTTGGTAT<br>TCAGCTGTATAATACCGGTTGTGCCTTTGGTATTGATCAGGTTTGTTATGGTCCGACACCGACC<br>GATACACCGGATTTTAGCGTTGCAATGGCAACCGATCTGCTGGAAAATTGGCCTGCAACCGTTA<br>ATGGTCGTCCGACCGGTTTTCAGCCGTATATTAGCTATCTGCGTCCGAGCCAGATTGTGATTGG<br>TTATCCGAGTCCGAATGCAAGCGGTGGTAGTGATGGTAGTCCGGTTACACCGACCACCACCATT<br>AAACGTGCAATTCAGTGTCTGAAAACAGCCATTGCAGGTAATACCAGCTGTGGTGTTTATGTTC<br>CGCCTCGTGCATATGGTAATATTGGTGGTGTGTTTAATTGGGAAGTGACCTACGATAAAAACAA<br>CCAGTTCAAATTCGCCAAAGAGCTGAAAAATTGCGCGATTAATGGCGTGTGTGAATAACTCGAG
CM13	ChiA-CTD E543M/ H544A mutant	CCATGGTCCATCATCATCATCATCATGTGGACGACGACGACAAGATGGTTACCGCAGCAAAAGG<br>TCGCATTATTGGTTATGTTCCAGGTTGGAAAACCCCTCCGGCAGCACAAGAACTGGCAAGCGCA<br>GGTTATACCCATGTTATGATTGCATTTGGTGTGTTCAGCACCAATACACCGGGTGTTATTGTTC<br>CGGCATTTGAAACCATTACCAAAGAGTATATTCAGAGCCTGCATCAGGCAGGCATTAAAGTTAT<br>TCTGAGCTTAGGTGGTGCACTGACCAGCATTCCGAATACCACCGTTGATTTTCATCAGGTTCTG<br>GTTGCAAGCAGCAGTCCGGAAGCATTTAAACAGACCTTTATTAACAGCCTGAAAGAACTGATTA<br>GCCAGTATGGTTTTGATGGCTTTGATACCGATATTATGGCGGGTATTAATGCCAGCGGTAGCTT<br>TAGCCAGCCGCAGGGTGATATTGCAGTTCTGGCAAGCATTATTAACACCATGTATAGCCAGAAT<br>AGCAGCCTGCTGATTACCCTGACACCGCAGGTTGCAAATATTGCAGCAACCAGCGGTTTTGATC<br>AGACCTGGGGTAATTATGCAAGCCTGATTATGCAGACCCATCAGAGTCTGGCATGGGTTGGTAT<br>TCAGCTGTATAATACCGGTTGTGCCTTTGGTATTGATCAGGTTTGTTATGGTCCGACACCGACC<br>GATACACCGGATTTTAGCGTTGCAATGGCAACCGATCTGCTGGAAAATTGGCCTGCAACCGTTA<br>ATGGTCGTCCGACCGGTTTTCAGCCGTATATTAGCTATCTGCGTCCGAGCCAGATTGTGATTGG<br>TTATCCGAGTCCGAATGCAAGCGGTGGTAGTGATGGTAGTCCGGTTACACCGACCACCACCATT<br>AAACGTGCAATTCAGTGTCTGAAAACAGCCATTGCAGGTAATACCAGCTGTGGTGTTTATGTTC<br>CGCCTCGTGCATATGGTAATATTGGTGGTGTGTTTAATTGGGAAGTGACCTACGATAAAAACAA<br>CCAGTTCAAATTCGCCAAAGAGCTGAAAAATTGCGCGATTAATGGCGTGTGTGAATAACTCGAG
CM14	ChiA-CTD E543M/ N547A mutant	CCATGGTCCATCATCATCATCATCATGTGGACGACGACGACAAGATGGTTACCGCAGCAAAAGG<br>TCGCATTATTGGTTATGTTCCAGGTTGGAAAACCCCTCCGGCAGCACAAGAACTGGCAAGCGCA<br>GGTTATACCCATGTTATGATTGCATTTGGTGTGTTCAGCACCAATACACCGGGTGTTATTGTTC<br>CGGCATTTGAAACCATTACCAAAGAGTATATTCAGAGCCTGCATCAGGCAGGCATTAAAGTTAT<br>TCTGAGCTTAGGTGGTGCACTGACCAGCATTCCGAATACCACCGTTGATTTTCATCAGGTTCTG<br>GTTGCAAGCAGCAGTCCGGAAGCATTTAAACAGACCTTTATTAACAGCCTGAAAGAACTGATTA<br>GCCAGTATGGTTTTGATGGCTTTGATACCGATATTATGCATGGTATTGCGGCCAGCGGTAGCTT<br>TAGCCAGCCGCAGGGTGATATTGCAGTTCTGGCAAGCATTATTAACACCATGTATAGCCAGAAT<br>AGCAGCCTGCTGATTACCCTGACACCGCAGGTTGCAAATATTGCAGCAACCAGCGGTTTTGATC<br>AGACCTGGGGTAATTATGCAAGCCTGATTATGCAGACCCATCAGAGTCTGGCATGGGTTGGTAT<br>TCAGCTGTATAATACCGGTTGTGCCTTTGGTATTGATCAGGTTTGTTATGGTCCGACACCGACC<br>GATACACCGGATTTTAGCGTTGCAATGGCAACCGATCTGCTGGAAAATTGGCCTGCAACCGTTA<br>ATGGTCGTCCGACCGGTTTTCAGCCGTATATTAGCTATCTGCGTCCGAGCCAGATTGTGATTGG<br>TTATCCGAGTCCGAATGCAAGCGGTGGTAGTGATGGTAGTCCGGTTACACCGACCACCACCATT<br>AAACGTGCAATTCAGTGTCTGAAAACAGCCATTGCAGGTAATACCAGCTGTGGTGTTTATGTTC<br>CGCCTCGTGCATATGGTAATATTGGTGGTGTGTTTAATTGGGAAGTGACCTACGATAAAAACAA<br>CCAGTTCAAATTCGCCAAAGAGCTGAAAAATTGCGCGATTAATGGCGTGTGTGAATAACTCGAG
CM15	ChiA-CTD E543M/ Q595A mutant	CCATGGTCCATCATCATCATCATCATGTGGACGACGACGACAAGATGGTTACCGCAGCAAAAGG<br>TCGCATTATTGGTTATGTTCCAGGTTGGAAAACCCCTCCGGCAGCACAAGAACTGGCAAGCGCA<br>GGTTATACCCATGTTATGATTGCATTTGGTGTGTTCAGCACCAATACACCGGGTGTTATTGTTC<br>CGGCATTTGAAACCATTACCAAAGAGTATATTCAGAGCCTGCATCAGGCAGGCATTAAAGTTAT<br>TCTGAGCTTAGGTGGTGCACTGACCAGCATTCCGAATACCACCGTTGATTTTCATCAGGTTCTG<br>GTTGCAAGCAGCAGTCCGGAAGCATTTAAACAGACCTTTATTAACAGCCTGAAAGAACTGATTA<br>GCCAGTATGGTTTTGATGGCTTTGATACCGATATTATGCATGGTATTAATGCCAGCGGTAGCTT<br>TAGCCAGCCGCAGGGTGATATTGCAGTTCTGGCAAGCATTATTAACACCATGTATAGCCAGAAT<br>AGCAGCCTGCTGATTACCCTGACACCGCAGGTTGCAAATATTGCAGCAACCAGCGGTTTTGATG<br>CGACCTGGGGTAATTATGCAAGCCTGATTATGCAGACCCATCAGAGTCTGGCATGGGTTGGTAT

TCAGCTGTATAATACCGGTTGTGCCTTTGGTATTGATCAGGTTTGTTATGGTCCGACACCGACC
GATACACCGGATTTTAGCGTTGCAATGGCAACCGATCTGCTGGAAAATTGGCCTGCAACCGTTA
ATGGTCGTCCGACCGGTTTTCAGCCGTATATTAGCTATCTGCGTCCGAGCCAGATTGTGATTGG
TTATCCGAGTCCGAATGCAAGCGGTGGTAGTGATGGTAGTCCGGTTACACCGACCACCACCATT
AAACGTGCAATTCAGTGTCTGAAAACAGCCATTGCAGGTAATACCAGCTGTGGTGTTTATGTTC
CGCCTCGTGCATATGGTAATATTGGTGGTGTGTTTAATTGGGAAGTGACCTACGATAAAAACAA
CCAGTTCAAATTCGCCAAAGAGCTGAAAAATTGCGCGATTAATGGCGTGTGTGAATAACTCGAG

*Restriction sites are underlined
